# A compendium of human genes regulating feeding behavior and body weight, its functional characterization and identification of GWAS genes involved in brain-specific PPI network

**DOI:** 10.1186/s12863-016-0466-2

**Published:** 2016-12-22

**Authors:** Elena V. Ignatieva, Dmitry A. Afonnikov, Olga V. Saik, Evgeny I. Rogaev, Nikolay A. Kolchanov

**Affiliations:** 1 Center for Brain Neurobiology and Neurogenetics, The Federal Research Center Institute of Cytology and Genetics of Siberian Branch of the Russian Academy of Sciences, Novosibirsk, 630090 Russia; 2 Novosibirsk State University, Novosibirsk, 630090 Russia; 3 Laboratory of Evolutionary Bioinformatics and Theoretical Genetics, The Federal Research Center Institute of Cytology and Genetics of Siberian Branch of the Russian Academy of Sciences, Novosibirsk, 630090 Russia; 4 Department of Systems Biology, The Federal Research Center Institute of Cytology and Genetics of Siberian Branch of the Russian Academy of Sciences, Novosibirsk, 630090 Russia; 5 BNRI, Department of Psychiatry, University of Massachusetts Medical School, Worcester, MA 15604 USA

**Keywords:** Feeding behavior, Body weight regulation, Brain, Network, PPIs, GWAS meta-analysis, Database

## Abstract

**Background:**

Obesity is heritable. It predisposes to many diseases. The objectives of this study were to create a compendium of genes relevant to feeding behavior (FB) and/or body weight (BW) regulation; to construct and to analyze networks formed by associations between genes/proteins; and to identify the most significant genes, biological processes/pathways, and tissues/organs involved in BW regulation.

**Results:**

The compendium of genes controlling FB or BW includes 578 human genes. Candidate genes were identified from various sources, including previously published original research and review articles, GWAS meta-analyses, and OMIM (Online Mendelian Inheritance in Man). All genes were ranked according to knowledge about their biological role in body weight regulation and classified according to expression patterns or functional characteristics. Substantial and overrepresented numbers of genes from the compendium encoded cell surface receptors, signaling molecules (hormones, neuropeptides, cytokines), transcription factors, signal transduction proteins, cilium and BBSome components, and lipid binding proteins or were present in the brain-specific list of tissue-enriched genes identified with TSEA tool. We identified 27 pathways from KEGG, REACTOME and BIOCARTA whose genes were overrepresented in the compendium. Networks formed by physical interactions or homological relationships between proteins or interactions between proteins involved in biochemical/signaling pathways were reconstructed and analyzed. Subnetworks and clusters identified by the MCODE tool included genes/proteins associated with cilium morphogenesis, signal transduction proteins (particularly, G protein–coupled receptors, kinases or proteins involved in response to insulin stimulus) and transcription regulation (particularly nuclear receptors). We ranked GWAS genes according to the number of neighbors in three networks and revealed 22 GWAS genes involved in the brain-specific PPI network. On the base of the most reliable PPIs functioning in the brain tissue, new regulatory schemes interpreting relevance to BW regulation are proposed for three GWAS genes (*ETV5, LRP1B*, and *NDUFS3).*

**Conclusions:**

A compendium comprising 578 human genes controlling FB or BW was designed, and the most significant functional groups of genes, biological processes/pathways, and tissues/organs involved in BW regulation were revealed. We ranked genes from the GWAS meta-analysis set according to the number and quality of associations in the networks and then according to their involvement in the brain-specific PPI network and proposed new regulatory schemes involving three GWAS genes (*ETV5*, *LRP1B*, and *NDUFS3*) in BW regulation. The compendium is expected to be useful for pathology risk estimation and for design of new pharmacological approaches in the treatment of human obesity.

**Electronic supplementary material:**

The online version of this article (doi:10.1186/s12863-016-0466-2) contains supplementary material, which is available to authorized users.

## Background

The pathogenesis of obesity is apparently complex. It involves multiple interactions among behavioral, environmental, and genetic factors. According to twin studies, 40–70% of inter-individual variability in body mass index (BMI), commonly used to assess obesity, is attributed to genetic factors [1–3].

Monogenic forms of obesity account for approximately 5% of severe obesity cases [4]. For most of the monogenic forms of obesity in murine models, human counterparts have been found. Eleven monogenic obesity genes have been identified to date [5]. Monogenic obesity results from mutations in genes involved in the hypothalamic appetite regulation system. Loss-of-function mutations causing deficiencies of appetite-regulating hormones or their receptors, such as leptin [6], leptin receptor [7], pro-opiomelanocortin (*POMC*) [8], and melanocortin 4 receptor (*MC4R*) [9], are examples of such monogenic syndromes. Furthermore, mutations in the proprotein convertase subtilisin/kexin type 1 (*PCSK1*) gene, a key component in the proteolytic processing of POMC, cause extreme childhood obesity and abnormal glucose homeostasis [10]. In addition, patients with chromosomal aberrations resulting in disruption or deletion of the single-minded homologue 1 gene (*SIM1*), which is essential for proper development of the paraventricular nucleus of hypothalamus, have early-onset obesity [11, 12]. Monogenic obesity also results from mutations in some other genes involved in eating behavior and energy balance regulation: (1) brain-derived neurotrophic factor (*BDNF*) [13]; (2) its receptor, tyrosine receptor kinase B (*NTRK2*) [14]; (3) SH2B adaptor protein 1 (*SH2B1*), involved in the regulation of leptin signaling [15, 16]; (4) *KSR2,* which encodes a scaffolding protein kinase suppressor of Ras 2, participating in signaling pathways relevant to glucose homoeostasis and food intake control [17]; (5) *TUB,* encoding Tubby bipartite transcription factor [18]. Although not formally defined as a syndrome, the clinical features of TUB deficiency in humans may be consistent with a novel ciliopathy [5]. Most monogenic obesity cases were investigated only in individual families; thus, their true contribution to “common” obesity in the general population is poorly known and, probably, underestimated [4].

A number of syndromes have been identified in addition to monogenic forms of obesity. The clinical features of these syndromes include obesity and developmental delay [5]. Examples are the Bardet–Biedl [19], Prader–Willi [20], and Alström [21] syndromes. These syndromes were earlier viewed as monogenic, but later studies pointed to a heterogeneous genetic background [4, 19, 20].

Genetic variants associated with “common polygenic obesity” were extensively sought in the pre-GWAS era using genome-wide linkage studies and candidate gene approaches. Unfortunately, none of the SNP markers discovered to have associations with common obesity had meaningful predictive power [4]. Many of them broadly varied across ethnic groups, and they were found difficult to replicate [22].

The development of high-throughput genotyping techniques in conjunction with the progress of statistical and computational methods and the completion of the HapMap and Human Genome Projects enable scientists to carry out large-scale genome-wide association studies, in which a large number of genetic variants are tested for association with a trait of interest [22]. Genome-wide association studies have identified multiple genetic variants associated with the risk of obesity or elevated BMI [23]. However, replication efforts very often yield very inconsistent results [24, 25]. It is important to determine how genetic variants influence body weight, but most of them are non-coding, and there is little understanding of how these variants contribute to BW control.

Recent publications on GWAS meta-analysis present current lists of lead SNPs (lead signals) and candidate genes (secondary signals), which were mostly revealed according to two main criteria: (1) the gene was the nearest to the index SNP; or (2) the gene was found in the vicinity of the lead SNP and was biologically related to obesity, a related metabolic disorder, or energy expenditure according to the results of manual literature mining [26–28]. In some other advanced GWAS meta-analysis reports [29, 30], additional and more sophisticated supportive data are considered, namely: (1) genes contain a cis-expression quantitative trait locus (eQTL) in linkage disequilibrium (LD) with the index SNP; (2) genes contain missense, or nonsense, or copy number variants; (3) genes have been prioritized by connections in published GRAIL (Gene Relationships Across Implicated Loci) abstracts; (4) genes have been prioritized by integrative methods implemented in the DEPICT tool [31]. Thus, GWAS papers register candidate genes, but biological functions of many of them revealed so far (especially for nearest genes or genes containing eQTL) remain unknown or poorly understood. Therefore, the explanation of biological functions of genes noted in GWAS in the context of BW regulation is a separate and essential task.

Thus, in spite of the huge body of information obtained by various experimental approaches, including genome-wide ones, the knowledge of the genetic prerequisites of obesity is insufficient.

The creation of the compendium of genes presumably involved in obesity can be based on the analysis of physiologic systems controlling BW, including basal metabolism, which is regulated by the nervous, endocrine, and immune systems.

The system controlling feeding behavior (FB) is among the most important ones, determining BW. It involves proteins and genes expressed in the brain [32] and in peripheral organs and tissues: the stomach, intestine, pancreas, and fat tissue. The central core of the system is formed by two types of neurons in the hypothalamic arcuate nucleus. They secrete neuropeptide Y (NPY) and the Agouti-related peptide (AgRP) (NPY/AgRP-expressing neurons) or α-melanocyte-stimulating hormone (α-MSH), which is produced from proopiomelanocortin (POMC) by proprotein convertases PCSK1 and PCSK2 (POMC-expressing neurons) [33]. The function of neurons of the arcuate nucleus is controlled by hormones (leptin, insulin, ghrelin, polypeptide YY (PYY), glucocorticoids, adrenocorticotropin, and the corticotropin-releasing hormone), as well as neurotransmitter systems of the brain (serotonergic, dopaminergic, adrenergic, and GABAergic), and neurotrophic factors (BDNF and others) [33, 34].

The objectives of this study were: (1) to compile a compendium of genes controlling human body weight and feeding behavior; (2) to construct and analyze networks formed by associations between genes/proteins from compendium; (3) to reveal tissues or organs, signaling or biochemical pathways, biological processes and physiological systems of the human body associated with genes from compendium; (4) to examine associations between genes identified by GWAS meta-analysis and other genes from compendium and to prioritize GWAS genes according to the number and quality of associations.

We compiled a compendium of 578 human genes collected from: (1) research and review articles, (2) OMIM, and (3) publications presenting GWAS meta-analysis results. We ranked all genes according to our knowledge about the biological roles of particular genes in BW and/or FB regulation. We found that considerable and overrepresented numbers of genes from the compendium encoded cell surface receptors, signaling molecules (hormones, neuropeptides, and cytokines), transcription factors, signal transduction proteins, and cilium and BBSome components or were present in the brain-specific list of genes expressed in a tissue-enriched manner. By using DAVID, we identified 27 pathways enriched in genes from the compendium that might be classified into the following categories: (1) *signaling molecules and interaction*; (2) *signal transduction*; (3) *endocrine system*; (4) *excretory system*; (5) *development*; (6) *endocrine and metabolic diseases*; (7) *tumors*. Module network analysis of the network involving homology or physical interactions between genes/proteins from compendium revealed notable clusters formed by G protein-coupled receptors and nuclear receptors, as well as clusters associated with cilium morphogenesis, transcription regulation, and insulin signaling.

We prioritized genes collected from the GWAS meta-analysis papers according to the number and quality of associations in the networks. We selected three most reliable PPIs that involved GWAS genes and proved to be functional in the brain tissue according to expression data. These PPIs involved three GWAS genes (*ETV5*, *LRP1B*, and *NDUFS3)* and four other proteins with known biological roles in BW and/or FB regulation. Then we manually reviewed literature related to these genes/proteins and constructed putative regulatory pathways implicating three selected PPIs. We hypothesized that physical interactions between ETV5 and AR, LRP1B and SERPINE1, NDUFS3, and ADRB2 might be regarded as potential mechanisms involving GWAS genes (*ETV5*, *LRP1B*, and *NDUFS3*) in the central regulation of body weight.

## Methods

### Extracting genes from diverse data sources. Scoring schemes

Genes were extracted from three data sources: (a) scientific publications (research papers and review articles), (b) the OMIM database, and (c) GWAS meta-analysis results presented in scientific papers (Table 1).

**Table 1:** The sources of data for creating the compendium of genes controlling human body weight and feeding behavior and characteristics of the corresponding gene sets

Source of data/description of the gene set	Short name of the data source and the gene set	Number of genes	Number of publications or database query
Research papers and review articles on FB-regulating genes	*Publications* (Additional file 1: Table S1)	105	17 review articles, 45 research papers
OMIM/genes possessing allelic variants associated with obesity, hyperphagia, or anorexia	*OMIM_allelic_variants* (Additional file 1: Table S2)	73	Command used in OMIM search: ‘hyperphagia’ OR ‘obesity’ OR ‘anorexia’ (Records with: gene map locus; Prefixes: +, *; Search in: allelic variants)
OMIM/terms *obesit*y, or *hyperphagia,* or *anorexia* were found in text fields (excluding the chapter devoted to allelic variants).	*OMIM_all_text* ^a^ (Additional file 1: Table S2)	263	Command used in OMIM search: ‘hyperphagia’ OR ‘obesity’ OR ‘anorexia’ (Records with: gene map locus; Prefixes: +, *; Search in: all text) *
OMIM and research papers/Genes whose mutant variants are implicated in the Bardet-Biedl and other syndromes associated with obesity.	*Syndromes* (Additional file 1: Table S3)	37	OMIM entries: 1) #209900; BARDET-BIEDL SYNDROME 1 (Genetic Heterogeneity of Bardet-Biedl Syndrome) 2) #176270; PRADER-WILLI SYNDROME 3) #203800; ALSTROM SYNDROME 4) #216550; COHEN SYNDROME 5) #103580; PSEUDOHYPOPARA-THYROIDISM, TYPE IA 6) #201000; CARPENTER SYNDROME 1 7) #147920; KABUKI SYNDROME 1; 8) #300867; KABUKI SYNDROME 2; 9) #157980; MOMO SYNDROME 10) #301900; BORJESON-FORSSMAN-LEHMANN SYNDROME 11) #182290; SMITH-MAGENIS SYNDROME 12) #180849; RUBINSTEIN-TAYBI SYNDROME 1 13) #612469; WAGRO SYNDROME; 3 review articles, 1 research paper
GWAS meta-analysis papers/genes located in the vicinity of a lead SNP	*GWAS meta-analysis* (Additional file 1: Table S4)	184	9 research articles

^a^The set *OMIM_all_text* included genes for which at least one of query terms was found in any chapter other than that on allelic variants but the latter contained none of the terms. If the query term was also found in the chapter on allelic variants, such gene was assigned to the set *OMIM_allelic_variants* and excluded from *OMIM_all_text*

The first data source included research papers and review articles describing genes that regulate FB in humans or in other mammalian species (mice or rats). This data source and the corresponding set of genes are designated below as *Publications*. In case the publication described a non-human mammalian gene, the homologous human gene was found and included into the compendium (provided with a special comment).

The second data source was An Online Catalog of Human Genes and Genetic Disorders, OMIM [35]. Three query terms (“obesity”, “hyperphagia”, or “anorexia”) were used. A total of 336 genes were extracted from OMIM. They were divided into two categories. The first category included 73 genes for which at least one of the query terms was found in the chapter dedicated to allelic variants. This data source and the corresponding set of genes are designated below as *OMIM_allelic_variants*. The second category included 263 genes for which at least one of query terms was found in any other chapter (outside the chapter on allelic variants). This data source and the corresponding set of genes are designated below as *OMIM_all_text*.

Since genes from the *OMIM_all_text* set were revealed in cases when query terms were sought through the whole text present in an OMIM entry, we consider such search process to be very similar to the text-mining approach. So genes from *OMIM_all_text* lacked data on allelic variants associated with pathological states, and they may be characterized as potential regulators of FB or BW.

OMIM was also used to extract genes implicated in the Bardet-Biedl and other syndromes associated with obesity. They are designated below as *Syndromes.* The list of syndromes associated with obesity and fitting genes was completed according to review articles devoted to the genetics of obesity as well [5, 36].

The third data source was scientific papers presenting GWAS meta-analysis results. We extracted 164 loci with genome-wide significant associations (*p*-value < 5 × 10^−8^) with BMI from nine articles. This data source and the corresponding set of genes are designated below as *GWAS meta-analysis*.

According to GWAS meta-analysis papers, each locus was characterized by a lead SNP and one or more genes located in the vicinity of the lead SNP. Consequently, we collected all genes mentioned in papers and provided each gene with comments regarding its status or potential significance: (1) biological candidate; (2) gene notable for biological relevance to obesity; (3) the BMI-associated variant is in strong linkage disequilibrium (LD; r2 ≥ 0.75) with a missense variant in the indicated gene; (4) eQTL; (5) nearest gene; (6) other nearby gene; etc. A total of 184 genes were obtained from the GWAS meta-analysis papers.

The procedure of ranking was performed on the base of our knowledge of the biological role of a gene in BW regulation. At this step, we divided genes into two groups. The first group (Rank_1: *genes with biological interpretation)* included all genes from the following data sources: (1) *Publications*, (2) *OMIM-allelic variant*, (2) *OMIM_all_text, (3) Syndromes*. Rank_1 also included some genes from the *GWAS meta-analysis* data source for which the biological role in FB or BW regulation had been described or explained in GWAS meta-analysis papers. The second group of genes (Rank_2: *genes without biological interpretation*) accumulated the rest of the genes from the *GWAS meta-analysis* gene set, whose biological functions had not been explained in the context of FB or BW regulation.

### Assignment of genes to functional categories and pathway analysis

To reveal protein-coding genes we used Gene_info table_2016_03_06 from EntrezGene (ftp://ftp.ncbi.nlm.nih.gov/gene/DATA/). To characterize the biological roles of protein-coding genes from the compendium, we divided them into nonoverlapping groups. Some groups (transmembrane receptors, signaling molecules (i.e. hormones, cytokines and neuropeptides), enzymes, etc.) were created manually. To select genes encoding transcription factors we used TFClass database [37]. To classify genes as a transcriptional regulators we used the list of 167 genes encoding proteins with chromatin-modifying activities that was compiled previously [38] from three databases: EntrezGene (http://www.ncbi.nlm.nih.gov/gene), CREMOFAC [39], and CR Cistrome [40]. To annotate genes encoding proteins related to the cilium or BBSome, we used genes extracted from EntrezGene utilizing the GO terms “BBSome” or “cilium” as a query. To confirm that some functional groups of genes were overrepresented in the compendium, we applied the web-based functional annotation tool known as the DAVID (Database for Annotation, Visualization and Integrated Discovery) tool [41]. The significance of GO terms was estimated through the adjusted *p-*values based on the Benjamini-Hochberg procedure (BH adjusted *p-*value), presented in the functional annotation chart (a built-in function of DAVID). The standard significance level 0.05 for BH adjusted *p-*value was applied.

Our second analysis was aimed at the identification of canonical pathways enriched in genes belonging to the compendium of genes regulating FB/BW. At this step, we also applied the DAVID tool [41]*.* Pathway enrichment analyses were undertaken for 459 genes from the list Rank_1: *genes with biological interpretation* and for four sets of genes — *Publications*, *OMIM_allelic_variants, OMIM_all-text, Syndromes, GWAS meta-analysis* (Table 1) - using DAVID against the ‘whole genome’ background. The enriched biological pathways from KEGG Pathway, Reactome and Biocarta databases are considered in our study. The significance of biological pathways was estimated through the BH adjusted *p-*value. Pathways with fold enrichments 1.5 or more and the BH adjusted *p-*values of at most 0.05 were considered interesting.

### Gene expression analysis

We invoked freely available data from the Human Protein Atlas [42] version 14 (http://www.proteinatlas.org/about/downloadrna_tissue.csv.zip) to classify genes from the compendium according to the tissue specificity of RNA and protein expression. This resource presents a classification of 19,709 protein-coding genes according to their tissue-specific expression into six categories (*Tissue enriched*, *Group enriched*, *Tissue enhanced*, *Expressed in all*, *Mixed*, *Not detected*), which are defined on the base of transcript levels in 32 human tissues. Our analysis included an additional category, “*Tissue elevated*”, comprising all genes assigned to the first three categories (*Tissue enriched*, *Group enriched*, and *Tissue enhanced*). The statistical significance of differences between the observed fractions of genes from the compendium classified into particular expression categories and the expected fraction was estimated by the Chi-square test. In each case, the expected fraction of genes calculated for a certain expression category was the same as the fraction of all protein-coding genes from the human genome that were ascribed to this expression category by [42].

At the next step, we used the TSEA (Tissue Specific Expression Analysis) tool (http://genetics.wustl.edu/jdlab/tsea/) to evaluate the significance of the overlap between genes from the compendium and the cell-specific lists of transcripts expressed in a tissue-enriched manner within a particular human organ or tissue. The TSEA tool [43] employed pSI statistics to determine tissue-enriched gene sets using publicly available RNA-seq data across the healthy, adult human body [44]. Within this approach, each cell type profile was compared to all other profiles and transcripts consistently enriched in each cell type were identified. For each transcript, the enrichment score (SI) was calculated and a pSI value was ascribed. Then cell-specific and enriched transcript lists were derived for each cell type at a given pSI threshold. The lower the pSI, the smaller, but more stringently specific, transcript lists were obtained. The TSEA tool accepts an input list of gene symbols and returns the enrichment analysis of their expressions across 25 tissues. As a result, candidate gene lists that overlap cell-specific lists of transcripts expressed in tissue-enriched manner in a particular tissue are identified by Fisher’s exact test with the Benjamini-Hochberg correction. To identify overrepresented lists of tissue-enriched genes and to reveal corresponding human organs or tissues, we set the pSI threshold to 0.05.

### Network construction

We employed the GeneMANIA Cytoscape plugin and STRING (Search Tool for the Retrieval of Interacting Genes/Proteins) to identify pairwise relationships among all genes/proteins from the compendium.

STRING [45] contains direct (physical) and indirect (functional) associations derived from different sources, including high-throughput experiments, co-expression, and prior knowledge. The associations of the following types were extracted from STRING in a tab-delimited format: (1) *Experimental*: protein-protein interactions, (2) *Homology:* homologous proteins, and (3) *Knowledge*: functional partners from pathways from the Nature Pathways Interaction Database (NCI) or KEGG. Pairwise relationships of all three types were filtered to include only high-confidence edges with STRING scores greater than 0.4.

We also used the GeneMANIA Cytoscape plugin [46] to identify pairwise physical relationships among genes from the compilation. Data were extracted in a tab-delimited format and filtered to include only edges with GeneMANIA weights greater than 0.01. To obtain a network of protein-protein interactions between objects (*Experimental*), data on pairwise physical relationships among genes extracted from STRING and GeneMANIA were imported into Cytoscape as two separate networks. Then the networks were merged, and duplicated edges were removed.

The other two networks (*Knowledge* and *Homology*) were obtained by importing into Cytoscape the associations of these two types extracted from STRING.

For each gene/protein the following additional data were imported into Cytoscape as attributes of nodes and used to arrange the visualization style: (1) the expression category of the gene (*Tissue enriched*, *Group enriched*, etc.); (2) the source of data (*Publications, OMIM_allelic_variants, OMIM_all_text, Syndromes, GWAS meta-analysis*); (3) knowledge on the biological role of the gene/protein (Rank_1: *genes with biological interpreta*tion or Rank_2: *genes without biological interpretation*).

### Module network analysis

The *Experimental* network was clustered using the ‘Molecular Complex Detection’ (MCODE) algorithm [47] with the default settings. MCODE is a Cytoscape plugin available via the Cytoscape plugin manager [48]. MCODE identifies discrete subnetworks (or clusters) from a larger network (e.g. STRING) and has the advantage over other clustering methods, as it allows direct fine tuning of clusters of interest without relying on the rest of the network.

The top three modules (clusters) of the *Experimental* network were screened under the conditions of minimum size = 4 and minimum score = 3.3. Then we expanded these three modules by adding the first neighbors of all nodes involved in each module.

To investigate the Gene Ontology (GO) functional enrichment for three expanded lists of genes we employed the DAVID tool. The enriched GO terms from the biological processes vocabulary GOTERM_BP_5 were considered in our study.

### Sublist of proteins expressed in brain

The list of genes important for central BW regulation was created by combining two groups of genes from the compendium. The first group included 93 genes revealed by TSEA (see *Gene expression analysis* section) as brain-specific at pSI threshold = 0.05. The second group included 203 genes whose expression in hypothalamic AGRP-expressing neurons or POMC-expressing neurons differed (with abs[log2(fold-change)] > 1) between mice fed ad libitum and deprived of food for 24 h (Suppl. Table 1 from [49], columns adj.lfc.agrp, adj.lfc.pomc, adj.lfc.AgPo). The two groups of genes were combined, and the resulting sublist of genes (designated below as *Brain-specific*) comprised 249 genes (Additional file 1: Table S6).

## Results

### The compendium of genes controlling human body weight and feeding behavior

We collected 578 genes from three sources (Table 1). Among them, 105 genes (*Publications*) were collected from scientific publications reporting the involvement of genes in FB regulation in humans, mice, or rats. (Additional file 1: Table S1). Association with monogenic non-syndromic obesity was found in 11 genes of the 105 [5]. We extracted 336 genes from OMIM using the query “*obesity”* OR *“hyperphagia”* OR *“anorexia”* (Additional file 1: Table S2). Of them, 73 genes (*OMIM_allelic_variants*) had OMIM-annotated allelic variants associated with FB abnormalities (hyperphagia, anorexia) or obesity. For the rest 263 genes (*OMIM_all_text*), the query terms «obesity», «hyperphagia», or «anorexia» were found in textual sections not related to allelic variants. The fourth gene set (*Syndromes)* was obtained from OMIM and scientific publications. It included 37 genes implicated in syndromes (Bardet-Biedl, Prader–Willi, Alstrom, etc.; 13 syndromes altogether) that included *obesity* as one of the phenotypic characteristics (Additional file 1: Table S3). The fifth gene set (*GWAS meta-analysis*) was collected from GWAS meta-analysis papers. It comprised 184 genes listed in articles and located in regions around 164 lead SNPs. The majority of lead SNPs (141 of 164, or 85%) were identified in European ancestry populations. The other 15% were found only in African, East Asian, Australian, and North American populations (Additional file 1: Table S4).

**Fig. 1:**
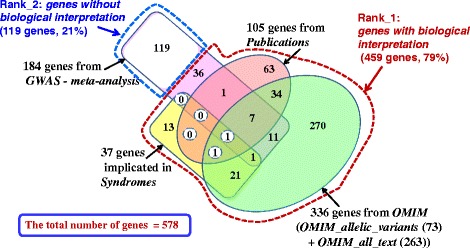
Venn diagram representing the numbers of genes in all gene sets *Publications*, *OMIM*, *Syndromes,* and *GWAS meta-analysis* used for creating the compendium of human genes controlling BW/FB. The red and blue dashed lines denote groups of genes obtained after ranking genes according to the knowledge of their biological role in body weight control

To determine the total number of genes relevant to BW regulation, we merged all gene sets: *Publications, OMIM_allelic_variants, OMIM_all_text, Syndromes,* and *GWAS meta-analysis*. With duplicates removed, a list comprising 578 unique genes was obtained (Fig. 1, Additional file 1: Table S5). We found that seven genes (*BDNF*, *MC4R*, *NTRK2*, *PCSK1*, *POMC*, *SH2B1, TUB*) were present in the following three gene sets: *Publications, OMIM, GWAS meta-analysis*. One gene (*BBS4*) was found at the intersection of three gene sets: *Syndromes, OMIM,* and *GWAS meta-analysis.* Thus, these eight genes found at the intersection of at least three gene sets may be considered the most significant for BW regulation.

### The ranking of genes according to the knowledge of their biological role in body weight control

According to the ranking procedure described in *Extracting genes from diverse data sources. Scoring schemes* section, all genes were divided into two groups: (1) Rank_1: *genes with biological interpretation*; (2) Rank_2: *genes without biological interpretation*. The first group (Rank_1) included all genes from the following four sets/informational sources: (1) *Publications,* (2) *OMIM_allelic_variants, (3) OMIM_all_text,* and *(4) Syndromes*. Some genes from *GWAS meta-analysis* data source were also included into Rank_1 if they were characterized in papers as (1) genes notable for their biological relevance to obesity or (2) biological candidates. Thus, 459 genes from the compendium (79% of the total number) were classified to Rank_1: *genes with biological interpretation* (Fig. 1). The other 21% of genes were classified to Rank_2: *genes without biological interpretation*. According to the ranking process, the second group of genes was composed entirely of genes from the *GWAS meta-analysis* data source.

### Functional composition of genes from the compendium

**Fig. 2:**
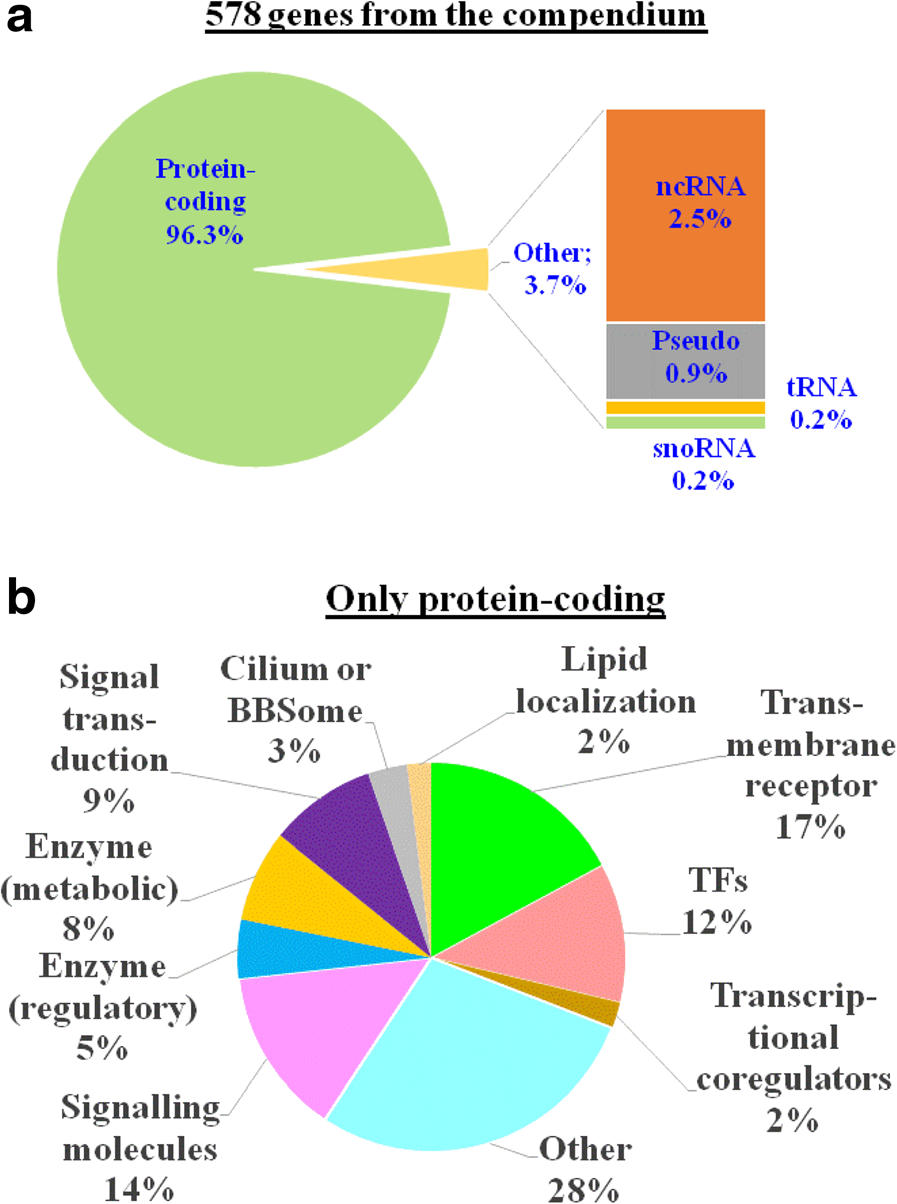
Distribution of functions of genes from the compendium. Panel **a** The fractions of protein-coding genes and other genes. Panel **b** Fractions of major functional groups of genes in the list of protein-coding genes

First, we determined the fraction of protein-coding genes in the compendium. It was 96.3% (Fig. 2a). The other 21 genes (3.7% of the total number) were non-protein-coding, with ncRNA (microRNA, antisense RNA, etc.) being the largest category. Genes encoding ncRNA comprised 2.5% of the total number of genes in the compendium. The largest portion of non-protein-coding genes were assigned previously to Rank_2: *genes without biological interpretation*. However, some non-protein-coding genes were categorized into Rank_1: *genes with biological interpretation*. Among them were *MIR148A*, *H19*, *LINC00237*, *MIR103A1*, *MIR107*, *MT-TK*, *NPY6R*.

Next, we divided the list of protein-coding genes into non-overlapping functional groups. We found that the five largest groups were: (1) transmembrane receptors (17% of genes from compendium); (2) signaling molecules (hormones, neuropeptides, cytokines, etc.) (14%); (3) transcription factors (12%); (4) and (5) metabolic and regulatory enzymes (8% and 5%, respectively) (Fig. 2b).

The official symbols of all genes included into the compendium, their data sources, molecular functions of encoded proteins, and Ranks are presented in Additional file 1: Table S5.

The hypothesis that the compendium was enriched in some functional groups of genes used in our classification (Fig. 2b) was tested with the DAVID tool. We performed GO analysis for 578 genes from the compendium and selected GO terms that characterized functional groups presented in Fig. 2b. We found that the compendium was enriched in genes associated with GO terms characterizing most groups: *transmembrane receptor activity, hormone activity, neuropeptide hormone activity, cytokine activity, transcription factor activity, signal transducer activity, lipid binding, cilium morphogenesis, BBSome* (Table 2). For these GO terms fold enrichments exceeded 1.5, and BH adjusted *p-*values were less than 0.05. According to GO analysis performed with DAVID, the other three of nine functional groups presented in Fig. 2b (*transcriptional coregulators, enzymes (regulatory),* and *enzymes (metabolic)*) were not enriched in the compendium.

**Table 2:** Overrepresented (BH adjusted *p*-value < 0.05) GO terms (biological processes, molecular functions, cellular compartments) that characterize functional groups of genes presented in Fig. 2b.

Functional group	GO category	GO class	Number of genes from the compendium annotated by the category	Fold Enrichment	BH adjusted *p-*value
Transmembrane receptors	GO:0004888 ~transmembrane receptor activity	Molecular function	89	2.09	1.6E-09
Transcription factors	GO:0003700 ~ transcription factor activity	Molecular function	52	1.63	1.1E-02
Signaling molecules (hormones, neuropeptides, cytokines, etc.)	GO:0005179 ~ hormone activity	Molecular function	37	10.48	1.5E-24
	GO:0005184 ~ neuropeptide hormone activity	Molecular function	15	19.95	7.6E-14
	GO:0005125 ~ cytokine activity	Molecular function	16	2.51	3.2E-02
Signal transduction	GO:0004871 ~ signal transducer activity	Molecular function	139	1.87	3.2E-12
Cilium or BBSome	GO:0060271 ~ cilium morphogenesis	Biological process	10	8.77	1.5E-05
	GO:0034464 ~ BBSome	Cellular component	7	31.56	1.8E-07
Lipid localization	GO:0008289 ~ lipid binding	Molecular function	34	2.31	3.9E-04
Transcriptional coregulators	None^a^				
Enzymes (regulatory)	None				
Enzymes (metabolic)	None				

The list of 578 genes from the compendium was analyzed with the DAVID-tool. The significance level for the BH adjusted *p*-value was 0.05

^a^None – No overrepresented GO terms were found

### KEGG pathway analysis

**Fig. 3:**
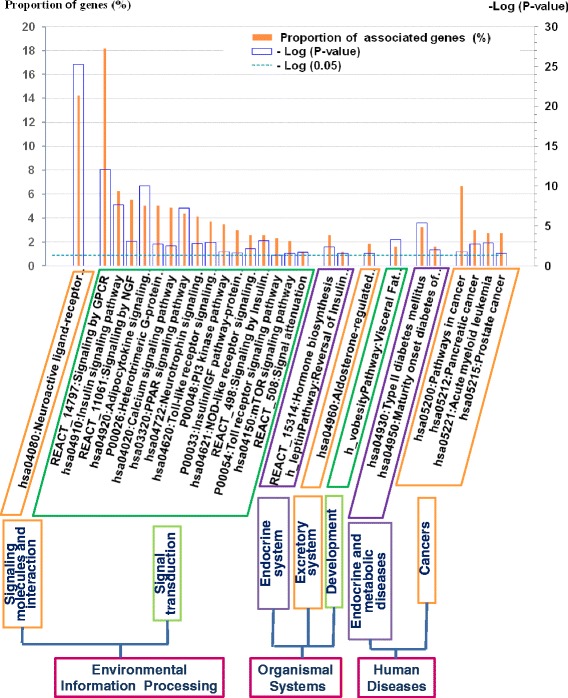
Association of genes from the compendium with major KEGG, REACTOME and BIOCARTA pathways. Pathways with fold enrichment > 1.5 and BH adjusted *p-*value < 5*10^−2^ are presented. Only genes from Rank_1: *genes with biological interpretation* were involved in analysis

To identify specific biological pathways that might be implicated in FB or BW control, we selected pathways overrepresented among genes from the compendium by applying the DAVID tool. The tool allows detection of enriched biological pathways or models for a variety of biological processes presented in the KEGG, REACTOME, BIOCARTA, and PANTHER databases. We suggested that a substantial part of genes from the *GWAS meta-analysis* set that had no interpretation (Rank_2: *genes without biological interpretation*) were not involved in the regulation of FB or BMI, so we excluded these genes from the pathway analysis. Just for this reason we analyzed the subset of 459 genes that had biological interpretations (Rank_1: *genes with biological interpretation*) at the first step. We identified 27 significantly enriched pathways or biological processes (Fig. 3). In all cases, the fold enrichment exceeded 1.5, and BH adjusted *p-*values were less than 0.05. We applied the hierarchical scheme provided by the KEGG pathway database to classify the enriched pathways into the following categories: (1) *signaling molecules and interaction*; (2) *signal transduction*; (3) *endocrine system*; (4) *excretory system*; (5) *development*; (6) *endocrine and metabolic diseases*; (7) *tumors*. The next hierarchical level of classification included such processes and entities as: (1) *Environmental Information Processing*, (2) *Organismal Systems*, and (3) *Human Diseases*.

We also performed pathway analyses for five sets of genes (*Publication, OMIM_allelic variant, OMIM_all_text*, *Syndromes, GWAS meta-analysis*) (Additional file 2: Figure S1). We found that the *Publications* set of genes had only six enriched pathways but the fractions of genes involved into two of them were very large (57% for *Signaling by GPCR* and 39% for *Neuroactive ligand-receptor interaction*). This observation points to a functional homogeneity of the *Publications* set and is in accordance with our previous observation that the compendium is enriched with transmembrane receptors and signaling molecules. Four enriched pathways were revealed for the *OMIM_allelic variants* set. In contrast, the *OMIM_all_text* set had 32 overrepresented pathways, and 12 of them (marked by plus signs in Additional file 2: Figure S1) were not overrepresented in the group of 459 genes that had biological interpretations (Rank_1: *genes with biological interpretation*). No enriched pathway was revealed for the very small set *Syndromes* (37 genes). The *GWAS meta-analysis* set also had only one enriched pathway (*Signalling by NGF*), comprising 9 genes (5% of the total number). The last observation points to high functional heterogeneity of genes from the *GWAS meta-analysis* set and confirms our guess that some genes from the set may have no relevance to FB or BW control.

### Gene expression analysis

We used two approaches to characterize the expression patterns of genes from the compendium.

**Fig. 4:**
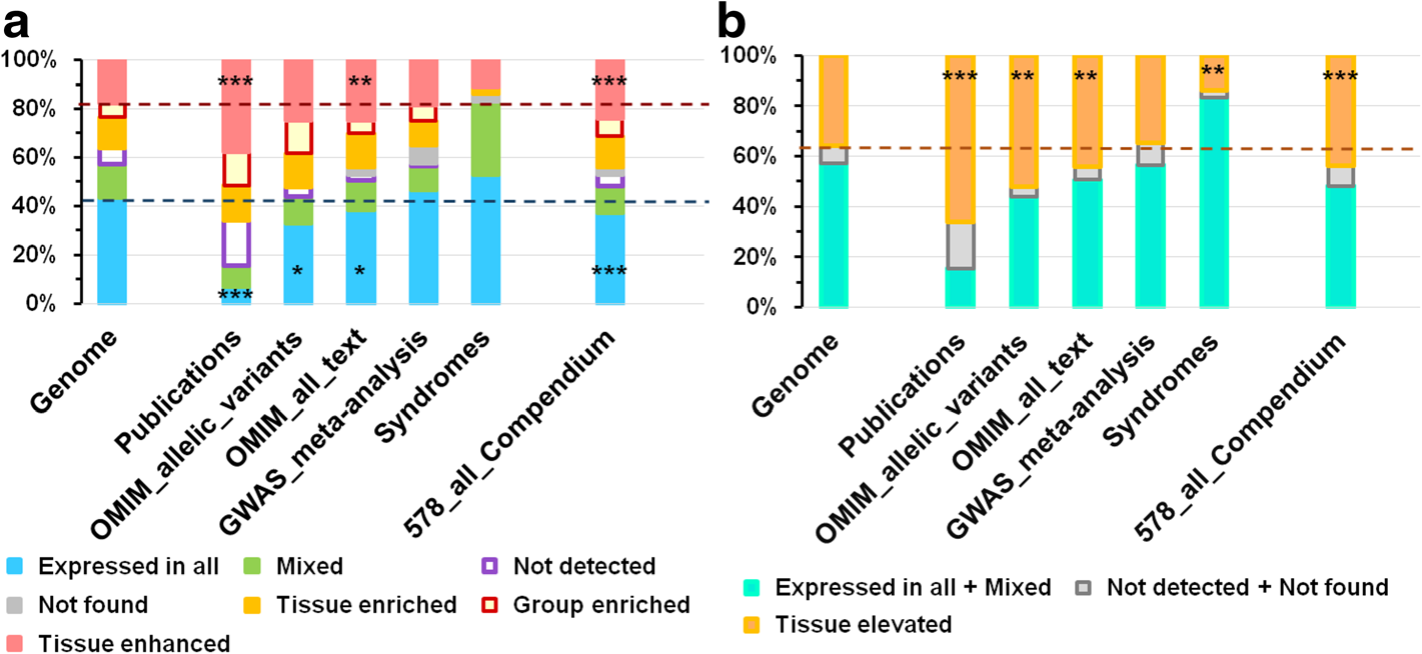
The fractions of genes classified according to tissue expression patterns and calculated for all protein-coding genes (*Genome*) or for all genes from the compendium (*578_all_Compendium*) and gene sets *Publications*, *OMIM_allelic_variants*, *OMIM_all_text, GWAS meta-analysis*, *Syndromes*. Panel **a** presents the fractions of genes classified into all six expression categories described in [42] and the category *Not found*. Panel **b** presents the fractions of genes belonging to three consolidated groups: (1) *Expressed in all* + *Mixed*; (2) *Not detected* + *Not found*; (3) *Tissue elevated*. The significances of the Chi-square test comparing the fractions of genes in test groups with the fractions in the whole-genome dataset are indicated with one (*p-*value < 0.05), two (*p-*value < 0.01), or three (*p-*value < 0.001) asterisks. The red and blue dotted lines in panel **a** and the orange dotted line in panel **b** denote the levels observed in the whole genome set of protein-coding genes

First, we divided genes from the compendium into six categories (*Expressed in all, Mixed, Not detected, Tissue enriched, Group enriched, Tissue enhanced*) basing on the classification of all human protein-coding genes presented in [42]. We observed differences in the distributions into categories between the whole genome set of protein-coding genes and genes from the compendium. The fraction of genes classified as *Expressed in all* decreased significantly (*p-*value < 0.001) in the compendium (Fig. 4a) as compared to the whole genome level (37.1% vs. 43.6%). In a good agreement with this observation, the compendium had an elevated (*p-*value < 0.001) fraction of genes from the *Tissue enhanced* category. The fraction of genes classified as *Tissue elevated* in the compendium was also higher (*p-*value < 0.001) than in the whole genome set (Fig. 4b). The enrichment in *Tissue elevated* genes was also found in sets of genes *Publications*, *OMIM_allelic_variants*, and *OMIM_all_text*. On the contrary, the set *Syndromes* was depleted of genes from the *Tissue elevated* category (*p-*value < 0.01). The most pronounced differences in expression patterns were observed between the whole genome set of protein coding genes and the *Publications* set. This set was depleted of genes classified as *Expressed in all tissues* at the significance level of 0.001 (Fig. 4a) and enriched (*p-*value < 0.001) in genes classified as *Tissue elevated* (Fig. 4b).

At the next step, we utilized the TSEA tool to identify overrepresented tissue-specific genes and to reveal corresponding tissues and organs. The same analysis was performed for the list of all 578 genes from the compendium (designated as *578_all_Compendium*) and seven subsets of genes (Rank_1: *genes with biological interpretation*, Rank_2: *genes without biological interpretation, Publications, OMIM_allelic_variants, OMIM_all_text, GWAS meta-analysis,* and *Syndromes*).

**Fig. 5:**
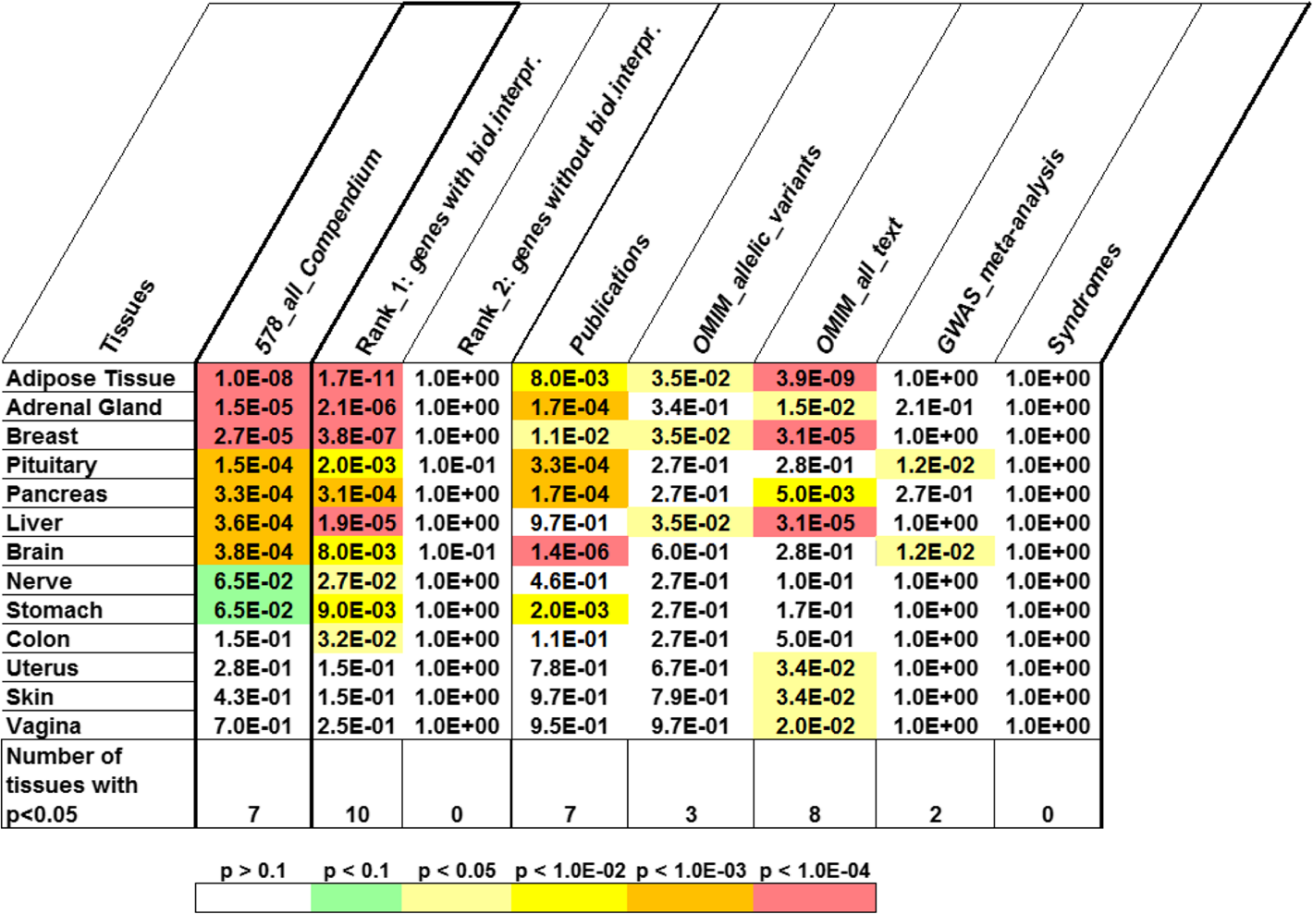
The heat map depicts the results of tissue-specific expression analysis performed with TSEA. Only tissues with overrepresented (*p-*value <0.05) cell-specific lists of tissue-enriched genes identified at the overlap with genes from the compendium or seven sets (Rank_1: *genes with biological interpretation,* Rank_2: *genes without biological interpretation, Publications, OMIM_allelic_variants, OMIM_all_text, GWAS meta-analysis,* and *Syndromes)* are shown. *P-*values derived by Fisher’s exact test with the Benjamini-Hochberg correction were obtained from the TSEA tool

**Fig. 6:**
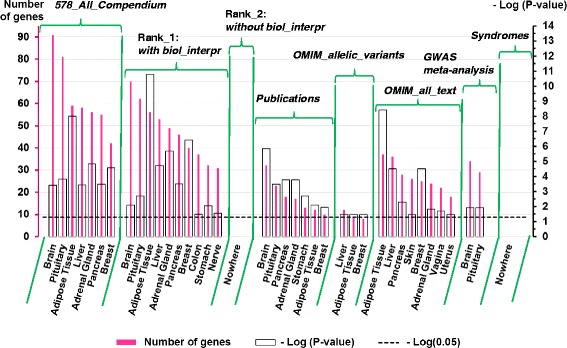
The number of genes from the compendium found with TSEA tool at the overlap with the cell-specific lists of transcripts expressed in a tissue-enriched manner. The lists of tissue-enriched transcripts were identified at pSI threshold = 0.05. Organs or tissues with Benjamini-Hochberg corrected *p-*values < 0.05 are presented

We found that genes from the *578_all_Compendium* list were overrepresented in tissue-enriched (pSI < 0.05) TSEA lists of genes expressed in seven tissues or organs (Fig. 5). The following tissues and organs were found: (1) adipose tissue and breast, two related tissues that store lipids; (2) the adrenal gland, pituitary gland, and pancreas, endocrine glands controlling metabolism via humoral signals; (3) the liver, central organ in lipogenesis, gluconeogenesis and cholesterol metabolism; (4) the whole brain, which performs the central regulation of feeding behavior - processing sensory signals (taste, olfactory, and food texture) and correlates them with other information. The highest number of genes from the set *578_all_Compendium* (93 genes) were found for the overlap with the brain list of tissue-enriched (pSI < 0.05) genes (Fig. 6).

The genes from seven gene sets had different numbers of tissues identified by TSEA (Fig. 5). Six tissues were identified for genes from the *Publications* set*.* The highest number of tissues (ten tissues) was identified by TSEA for the group of genes Rank_1: *genes with biological interpretation.* The group of genes Rank_2: *genes without biological interpretation,* which, in our opinion, contained a large portion of genes unrelated to BW regulation, had no tissues identified by TSEA. Seven, three, eight, and two tissues were found for sets *Publications*, *OMIM_allelic_variants, OMIM_all_text*, and *GWAS meta-analysis,* respectively. No tissue was identified by TSEA for the set *Syndromes* at any pSI threshold. Thus, TSEA identified at least two tissues for five out of seven sets examined (Fig. 5). We found that for three of these five sets of genes (Rank_1: *genes with biological interpretation, Publications*, and *GWAS meta-analysis*) the highest numbers of genes were found at the overlaps with the brain list of tissue-enriched genes compiled by TSEA tool at the pSI threshold = 0.05 (Fig. 6).

### Networks formed by associations between genes/proteins

To obtain more characteristics of functional systems involved in the regulation of body weight, we reconstructed networks presenting pairwise interactions between genes/proteins. Data on pairwise interactions were obtained from STRING [45] and GeneMANIA [46] (see *Network construction* section) and uploaded into Cytoscape [48]. Thus, three networks comprising interactions of three different types (*Experimental, Knowledge,* and *Homology*) were constructed and analyzed with Cytoscape and its plugin MCODE.

**Fig. 7:**
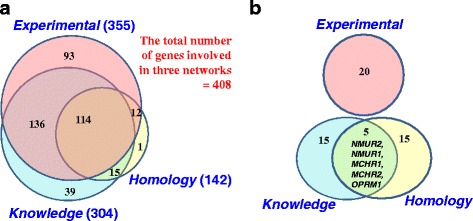
Venn diagrams representing the numbers of genes involved in three networks *Experimental*, *Knowledge,* and *Homology*. Panel **a** shows the total number of genes in each network. Panel **b** the lists of 20 genes that had the highest numbers of neighbors in each network

The maximal number of edges and the maximal average number of neighbors were found in the *Knowledge* network (Table 3). The maximal number of nodes was found in the *Experimental* network. None of the three networks involved all the 578 genes from the compendium (Fig. 7a, Table 3. We found that the PPI network (*Experimental*) involved the maximal portion of all genes from the compendium (62%). The *Knowledge* and *Homology* networks contained 53% and 25% of the total number of genes, respectively. Altogether, all the three networks included 71% of the total number of genes in the compendium (408 out of 578) (Fig. 7a).

**Table 3:** Characterization of three networks formed by associations between genes/proteins from the compendium

Network/Association type	Number of nodes (genes/proteins)/Number of genes as a percentage of all genes from the compendium	Number of edges	Average number of neighbors	Genes with the highest numbers of neighbors
*Experimental*	355 (62%)	1254	4.3	*ESR1, SIRT1, AR, NFKB1, STAT3, MAPK3, HDAC3, UBB, PTPN11*
*Knowledge*	304 (53%)	2403	15.9	*AGT, KNG1, MCHR1, PMCH, NMUR2, NMUR1, NMS, NMU, MCHR2, POMC*
*Homology*	142 (25%)	522	7.4	*NMUR2, NMUR1, MCHR1, MCHR2, OPRD1, NPY1R*
*All three types*	The total number of genes involved in all three networks = 408 (71%)

Genes involved in each network were ranked according to the number of neighbors, and the lists of 20 top genes were formed (Additional file 1: Table S7). The first genes were *ESR1* in the network *Experimental* (43 neighbors)*, AGT* (77 neighbors) and *NMUR2* (36 neighbors) in the *Knowledge* and *Homology* networks respectively. The intersections between three lists containing top 20 genes that had the maximal numbers of links in each network were analyzed (Fig. 7b). The portion of shared genes was very low. We found only five shared genes at the intersection of the *Knowledge* and *Homology* lists of top 20 genes.

We found that three lists of top 20 genes were different with respect to their expression patterns, data sources, and functions (Additional file 1: Table S7):

- In the network *Experimental*, most of the top genes (15 genes out of 20) were classified according to data from the Human Protein Atlas [42] as *Expressed in all.* Sixteen of the twenty genes belonged to the *OMIM_all_text* gene set.
- In the network *Knowledge*, a considerable portion of genes (13 out of 20) belonged to the *tissue-elevated* category (see *Gene expression analysis* section). The genes were included into the compendium mostly on the base of *Publications* (12 genes) and *OMIM_all_text* (9 genes) data sources.
- In the network *Homology*, 13 out of 20 genes were from the *tissue-elevated* category (see *Gene expression analysis* section) and six genes were classified as *Not detected*. Eighteen of twenty genes were from the set *Publications*. All twenty genes encoded G protein–coupled receptors.

We found that the lists of genes involved in three reconstructed nets (*Experimental, Knowledge and Homology*) overlapped to a certain extent (Fig. 7a). The merged list of genes involved in all three networks examined contained 408 genes (71% of the total number), which was more than the amounts of genes in each of the three separate networks (Table 3). This fact and the very small fraction of shared genes for three lists of 20 top genes (Fig. 7b) motivated us to consider networks of all three types in our further analysis.

### Module network analysis

**Fig. 8:**
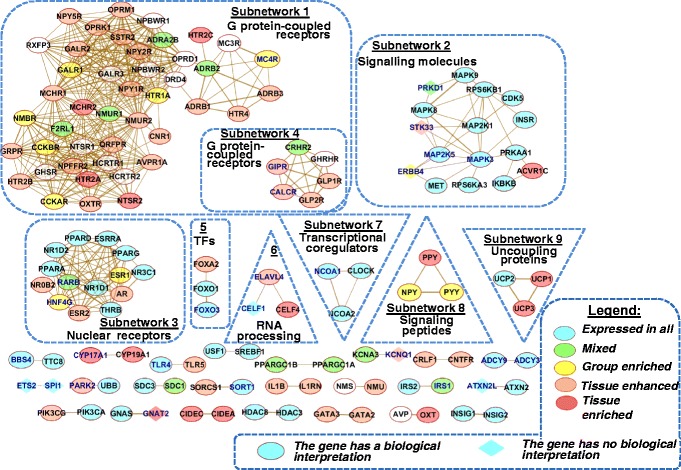
Unconnected subnetworks from the network formed by associations between homologous proteins (*Homology*). The colors of nodes indicate expression categories of genes (see legend) assigned according to data from the Human Protein Atlas [42] (see *Gene expression analysis* section). Thicker lines represent the stronger associations. Names of genes/proteins from the *GWAS meta analysis* set are shown in blue. Subnetworks with three or more nodes are outlined by dotted line. TFs – transcription factors

To explore groups of homologous proteins, a network formed by associations between homologous proteins extracted from STRING database was examined. This network included several unconnected subnetworks (Fig. 8). Two subnetworks (*Subnetwork 1*, 44 genes, and *Subnetwork 4*, 6 genes) contained G protein–coupled receptors. *Subnetwork 2* (17 genes) was formed by signal transduction proteins (Mitogen-activated protein kinases, other kinases, and some other proteins, such as INSR or IKBKB). *Subnetwork 3* (14 genes) included transcription factors from the nuclear receptor superfamily. Each of the next five subnetworks (from fifth to ninth) involved three proteins. Other 24 unconnected subnetworks contained two proteins each (Additional file 1: Table S8).

**Fig. 9:**
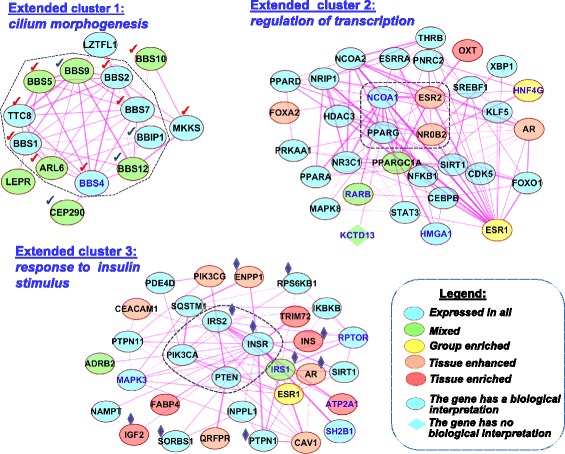
Three extended clusters revealed in the network *Experimental* formed by physical interactions between proteins from the compendium. Dashed lines denote the initial three clusters comprising 10, 4, and 4 proteins, which were identified with the MCODE tool. For Cluster 1: red check marks denote nine proteins annotated by GO term *cilium morphogenesis;* four genes marked by blue check marks are localized in primary cilia according to [56]. For Cluster 3: blue lozenges mark proteins associated with GO term *response to insulin stimulus*. Thicker lines represent stronger associations. The color legend and other designations follow Fig. 8

To characterize the functional domains of the network formed by physical interactions between proteins, we explored clusters identified by the MCODE tool (see *Module network analysis* section). Three clusters with node numbers exceeding 3 and scores exceeding 3.3 were found. As the numbers of nodes in each of these clusters were not large (10, 4, and 4 nodes), we identified the first neighbors for all genes from the clusters and included them into extended groups of genes. By this means, we obtained three extended clusters comprising 15, 33, and 31 nodes (Fig. 9).

Taking into account the classification of protein-coding genes according to their tissue-specific expression available from the Human Protein Atlas [42], we observed the expression patterns of genes involved in all three extended clusters. We found that the largest fractions of genes involved in the three extended clusters (100, 79, and 61%, respectively) were classified as *expressed in all* or *mixed*.

Then three extended groups/lists of genes were subjected to Gene Ontology (GO) functional enrichment analysis (see *Module network analysis* section), and it was found that (1) extended group 1 (extended cluster 1) was enriched in genes involved in *cilium morphogenesis*; (2) 85% of genes from extended group 2 (extended cluster 2) were related to the *regulation of transcription*; (3) ten proteins from extended group 3 (extended cluster 3), including two of four proteins from initial cluster 3 (IRS2, INSR), were involved in *response to insulin stimulus* (Fig. 9, and Additional file 1: Table S9).

### The ranking of genes from the GWAS meta-analysis set on the base of their weights (numbers of neighbors) in the networks

**Fig. 10:**
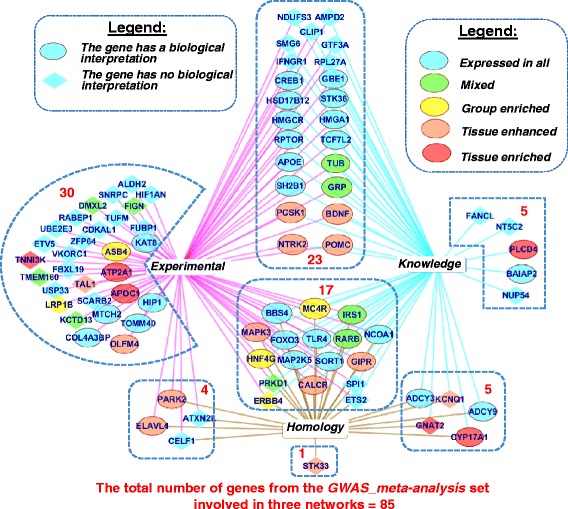
Genes from the *GWAS meta-analysis* set that are involved in three networks (*Experimental, Knowledge* and *Homology*). Red numerals indicate the numbers of genes that were found in three, two, or one networks. The colors of nodes correspond to RNA expression categories according to data obtained from the Human Protein Atlas [42] (see *Gene expression analysis* section). An edge width is proportional to the number of neighbors for the corresponding individual gene/protein in each network

To obtain additional characteristics for genes from the *GWAS meta-analysis* set, we considered their links in three networks that were described previously. In the *Experimental* network we found 74 genes/proteins from the *GWAS meta-analysis* set that had at least one neighbor. The greatest numbers of the first neighbors were possessed by MAPK3 (22 neighbors), IRS1 (17 neighbors), and NCOA1 (16 neighbors) (Additional file 1: Table S10). In the *Knowledge* and *Homology* networks, we found, respectively, 50 and 27 genes/proteins from the *GWAS meta-analysis* set that had at least one neighbor. The highest numbers of the first neighbors in the *Knowledge* network were found for POMC (66 neighbors), ADCY3 (65 neighbors) and ADCY9 (64 neighbors). In the *Homology* network, the three top genes/proteins *—* HNF4G, MAPK3, RARB — had 12, 11, and 11 first neighbors, respectively. Eighty-five genes/proteins from the *GWAS meta-analysis* set (~46% of the total number) had at least one first neighbor in at least one of the three networks examined (Fig. 10). Forty of them were from the group Rank_2: *genes without biological interpretation.* In Fig. 10 (and in all figures below), genes from the group Rank_2: *genes without biological interpretation* are enclosed in lozenges.

### Detection of GWAS genes in the Brain-specific sublist

**Fig. 11:**
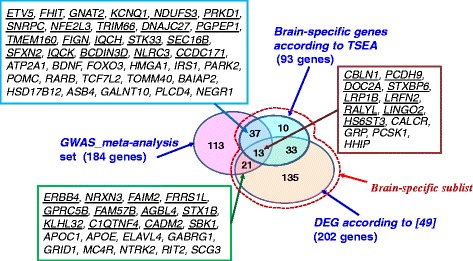
Venn diagram representing intersections between the *GWAS meta-analy*sis set and two groups of genes *Brain-specific gene according to TSEA* and *DEG according to* [49] that gave rise to the *Brain-specific* sublist of genes from the compendium (see *Sublist of proteins expressed in brain* section). Callout rectangles show genes that were found at the intersections of the gene set and two gene groups. Genes belonging to the group Rank_2: *genes without interpretation* are underlined

First, we included the annotation of two new characteristics of genes from the compendium into Additional file 1: Table S5: (1) *Brain-specific gene according to TSEA* for 93 genes that, according to TSEA, belonged to the cell-specific list of transcripts enriched in brain at the pSI threshold = 0.05; (2) *differentially expressed gene (DEG) according to* [49] for 203 genes found to be differently expressed in hypothalamic AGRP- or POMC-expressing neurons of mice either fed ad libitum or deprived of food. We found that 45 of all 578 genes in the compendium belonged to both categories, and among them 13 genes were from the *GWAS meta-analy*sis set (Fig. 11). Nine of these 13 GWAS genes were from Rank_2: *genes without interpretation*: *CBLN1, PCDH9, DOC2A, STXBP6, LRP1B, LRFN2, RALYL, LINGO2,* and *HS6ST3*. A total of 71 GWAS genes were found to be differentially expressed according to [49] or to be in the TSEA cell-specific list of transcripts enriched in brain at pSI threshold = 0.05. Among these 71 GWAS genes, 34 genes were annotated as *Brain-specific gene according to TSEA* and 50 genes were annotated as DEGs according to [49] (see Additional file 1: Table S5).

### Subnetwork involving proteins expressed in brain: revealing GWAS genes

**Fig. 12:**
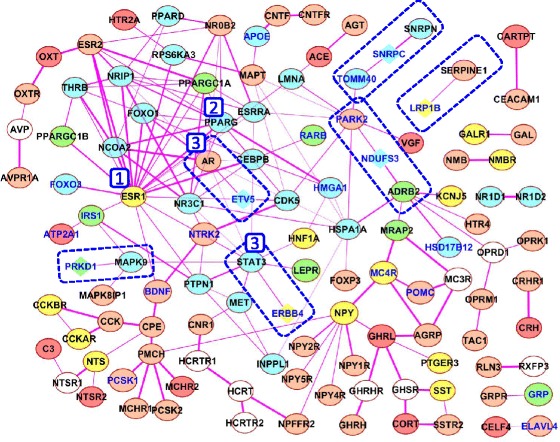
The *Experimental_brain-specific* network formed by physical interactions between genes/proteins from the sublist *Brain-specific* (see *Sublist of proteins expressed in brain* section). Ellipses denote proteins/genes from the Rank_1: *genes with biological interpretation* group, lozenges denote proteins/genes from Rank_2: *genes without biological interpretation.* Names of genes/proteins from the *GWAS meta analysis* set are shown in blue. Dashed rectangles denote associations that involve genes/proteins from the group Rank_2: *genes without biological interpretation.* Blue numerals denote the ranks of nodes calculated according to their weight (the number of first neighbors). The color legend and other designations are the same as in Figs. 8, 9, and 10

At the next step of our study, we reconstructed a network formed by physical interactions between proteins/genes annotated in the Additional file 1: Table S5 as *Brain-specific gene according to TSEA* or *DEG according to* [49] (see also *Brain-specific* sublist, Additional file 1: Table S6). This network was designated *Experimental_brain-specific*. It included 117 proteins and 172 associations between them (Fig. 12). All nodes in the network were ranked according to their degree (number of neighbors). The highest degree (20 neighbors) was found for estrogen receptor 1 (ESR1). This protein was assigned rank one (Additional file 1: Table S11). Rank two was assigned to PPARG, which had 11 neighbors, and rank three was shared by AR and STAT3, each having 10 neighbors. The *Experimental_brain-specific* network involved 22 genes from the *GWAS meta-analysis* set, and six of them were from Rank_2: *genes without biological interpretation*. Two of these six proteins (NDUFS3, SNRPC) had two neighbors and other four proteins (ERBB4, ETV5, PRKD1, LRP1B) were found to have one neighbor each (Table 4). The numbers of first neighbors ranged from high for AR and STAT3 (both had 10 neighbors) to medium for PARK2 and ADRB2 (8 and 7 neighbors) and low for MAPK9, TOMM40, SERPINE1, and SNRPN (4, 2, 1, and 1 neighbors respectively).

**Table 4:** Genes from the *GWAS meta-analysis* set involved in the *Experimental_brain-specific* network that were classified into Rank_2: *genes without biological interpretation* and their first neighbors

Genes from the *GWAS meta-analysis* set				First neighbor(s)			Association	
Gene	RNA tissue category/DEG^a^or TSEA gene^b^	GWAS meta-analysis paper	Lead SNP	Gene/Data source	Number of neighbors/Rank^c^	RNA tissue category/DEG^a^ or TSEA gene^b^	Quality of evidence	Comment
*ETV5*	*Expressed in all*/DEG	[30] [29]	rs9816226 rs1516725	*AR*/*OMIM_all_text*	10/3	*Expressed in all*/DEG	Very high	ETV5 (ERM) and AR were found interacting in humans [91]
*LRP1B*	*Group enriched*/DEG and TSEA	[30] [29]	rs2890652 rs2121279	*SERPINE1*/*OMIM_all_text*	1/12	*Tissue enhanced*/DEG	Very high	LRP1B and SERPINE1(=PAI-1) were found interacting in humans [96].
*NDUFS3*	*Expressed in all*/DEG	[30]	rs3817334	*ADRB2*/*OMIM_allelic _variants* *PARK2*/*GWAS meta-analysis*	7/6 8/5	*Mixed*/DEG *Tissue enhanced*/DEG	Very high Medium	NDUFS3^d^ and ADRB2 were found interacting in humans [103]. Human NDUFS3 interact with PARK2 through one intermediate protein [104].
*SNRPC*	*Expressed in all*/DEG	[29]	rs205262	*TOMM40*/*GWAS meta-analysis* *SNRPN*/*Syndrome*	2/11 1/12	*Expressed in all*/DEG *Expressed in all*/TSEA	Medium Medium	Interaction in humans has been curated in BioGRID interaction database on the base of [105], but it was not found in the main text of the article or in Suppl. files Putative homologs of human SNRPC and SNRPN were found interacting in yeast [106]
*ERBB4*	*Group enriched*/TSEA	[29]	rs7599312	*STAT3*/*OMIM_all_text*	10/3	*Expressed in all*/DEG	Medium	Putative homologs (ErbB-2 and Stat3) were found interacting in mice [107]
*PRKD1*	*Mixed*/DEG	[30] [29]	rs11847697 rs12885454 rs11847697	MAPK9/*OMIM_all_text*	4/9	*Expressed in all*/TSEA	Medium	Human PRKD1 (=PKD) and MAPK8 (=JNK), which is MAPK9 homolog, were found interacting in humans [108]

^a^ DEG, differentially expressed gene in mouse hypothalamic AGRP- or POMC-expressing neurons according to [49]

^b^ TSEA gene. Revealed by TSEA (see *Gene expression analysis* section) as brain-specific at the pSI threshold = 0.05

^c^ The rank was defined for each gene in the network *Experimental_brain-specific* on the base of the number of first neighbors

^d^ In [103] NDUFS3 was noted as a precursor of NADH-ubiquinone oxidoreductase flavoprotein 3 isoform a (see Supplemental data in [103])

We also found that 132 genes from the compendium were present in the *Brain-specific* sublist (Additional file 1: Table S6), but these genes/proteins were not associated with any other nodes (genes/proteins) in the *Experimental_brain-specific* network (In Additional file 1: Table S11, these genes are presented in part (B) and are denoted as *isolated* (*single)* nodes. We found that 49 genes denoted as *isolated* nodes were from the *GWAS meta-analysis* set. Among them, 36 genes were from Rank_2: *genes without biological interpretation*.

Using the PubMed links presented by STRING and GeneMANIA, we checked pieces of evidence confirming associations between proteins and found that three associations (between ETV5 and AR, LRP1B and SERPINE1, NDUFS3 and ADRB2) were of very good quality. In Table 4, the quality of proofs for these three associations is denoted as *Very high*. Other associations were confirmed by interactions between homologous proteins in other species (SNRPC and SNRPN) or interactions with paralogous protein in humans or other species (ERBB4 and STAT3, PRKD1 and MAPK9), or even interactions through one intermediate protein (between NDUFS3 and PARK2). In Table 4, the qualities of these proofs are denoted as *High or Medium*.

## Discussion

### A compendium of human genes regulating FB and BW

To obtain a systematic overview of genes controlling human body weight and feeding behavior, which may serve as therapeutic targets, we created a compendium of genes relevant to the impairment of feeding behavior and elevated body mass index. At present, the compendium contains information about 578 genes, for which there are indications of their direct or indirect relevance to FB or BW regulation (Fig. 1). We did not find any analogs for such a comprehensive gene catalog in publications. A catalog of eleven monogenic obesity genes identified to date and 227 genetic variants associated with polygenic obesity was presented in [5]. Ninety-seven GWS loci associated with BMI obtained from meta-analysis of nearly 340,000 individuals were described in [29]. A list of 38 genes used in commercially available nutrigenomic tests was compiled in [50]. Thirteen of these 38 genes (*HMGCR, APOE, APOB, AGT, CRP, ADRB1, TNF, APOA5, LPL, ADRB3, ADRB2, COMT,* and *UCP2*) are present in the compendium described in the current study. The results of nutrigenomics research studies of the 38 genes were subsequently meta-analyzed in [50] to identify possible associations between the genes of interest and dietary intake and/or nutrient-related pathologies. No specific or statistically significant association were identified for any of the 38 genes of interest. The authors made a conclusion that the need for thorough and continuous nutrigenomics research was evident as it would be a highly promising tool in precision medicine. The application of genomic information in the context of nutritional choice requires the continuing education of healthcare professionals [51] and creation of new evidence evaluation by test providers [52]. Therefore, we consider studies presenting compilations of genes and their analysis useful in this context.

### Classification and functional analysis

On the base of our knowledge of the biological role of genes in BW regulation we classified all genes from compendium into two groups: (1) Rank_1: *genes with biological interpretation,* which included 79% of genes in compendium; and (2) Rank_2: *genes without biological interpretation*, which included the remaining 21% genes (Fig. 1). At the final steps of our study, we analyzed PPIs involving some genes from the group Rank_2 and hypothesized their potential role in BW regulation (*The ranking of genes from the GWAS meta-analysis set on the base of their weights (numbers of neighbors) in the networks* and *Detection of GWAS genes in the Brain-specific sublist* sections).

Protein-coding genes constituted 96.3%, the overwhelming majority of genes in the compendium (Fig. 2a). Among them, six major overrepresented functional groups were found: (1) transmembrane receptors; (2) transcription factors; (3) signaling molecules (hormones, neuropeptides, cytokines); (4) signal transducers; (5) cilium and BBSome components; (6) lipid binding proteins (Table 2). Thus, we obtained an updated list of molecular functions important for BW regulation. The significance of these molecular functions for FB and BW regulation is obvious. The key role in regulation of FB and energy balance belongs to the central nervous system, being implemented through a complex interplay among neurons. This interplay is supported by neurotransmitters (neuromediators, neuropeptides, and releasing factors), which modulate neuronal activity via interactions with cellular transmembrane receptors [33, 53, 54]. In turn, receptors activate signal transducers, which effect transcription factor activity [55]. Cilia transduce intracellular signaling activated in response to various homeostatic neuropeptides, neurotransmitters, and hormones (such as neuropeptide Y, melanin-concentrating hormone, insulin, leptin, etc.) [56, 57]. Lipid binding proteins are involved in lipid transport and metabolism [58–60].

To evaluate the degree to what specific biochemical/signaling pathways or biological processes might be involved in FB or BW regulation, we attempted to define overrepresented pathways from the KEGG, REACTOME, and BIOCARTA databases with the DAVID tool. The benefits of using the ontological and pathway analyses for functional annotation of group of genes revealed by different criteria have been considered in numerous publications [38, 61, 62]

We identified a broad variety of enriched pathways/processes. Twenty-seven overrepresented pathways were found for the group Rank_1: *genes with biological interpretation* (Fig. 3), and the total number of overrepresented pathways found after analysis (Additional file 2: Figure S1) performed for additional five gene sets (*Publications*, *OMIM_allelic_variants*, *OMIM_all_text*, *Syndromes* and *GWAS meta-analysis*) was 44. This result is in good agreement with the notion that the process of body weight regulation is very complex [29, 63–65]. Pathway analysis provided evidence that elevated BMI might result from abnormalities in a large number of particular cellular or organismal processes (*signaling molecules and interaction*, *signal transduction*, or *development*) and organismal systems (*endocrine or excretory system*).

It is also important to keep in mind that most pathway models are not exclusive, i.e., they show considerable overlaps, reflecting the frequently observed synergy in signaling [66]. Such overlaps are most frequently observed for (1) signal transduction pathways, which often share a set of protein kinases, and (2) specific types of cancers sharing signal transducers like STAT3, VEGFB, NFKB1, IKBKB, TGFB1, PIK3CA, MAP2K1, MAPK3, MAPK8, MAPK9, etc. We think that it was a redundancy of signal transduction pathways annotated in any of the three database used (KEGG, REACTOME BIOCARTA) and a redundancy of pathways involved in specific types of cancer in KEGG pathway database that predetermined a considerable abundance of enriched pathways related to *signal transduction* and *cancers* revealed by DAVID.

Nevertheless, we propose that all enriched pathways be kept in mind as models of biological processes relevant to body weight regulation. We think that in any case their consideration may be useful for designing new pharmacological approaches for the treatment of BW abnormalities.

### Gene expression analysis

Tools such as DAVID, PANTER, REVIGO, and others [65, 67, 68] are a rich source of functional data, but they are static resources that rely on manually curated information from GO, KEGG PATHWAY, Reactome Pathway, Biocarta databases, etc. That is why we think that the results of pathway analysis performed with DAVID (or other similar tools) are not completely objective or unbiased. For this reason, in order to obtain additional functional characteristics of genes from the compendium, we utilized another approach, based on a dynamic source of information, such as gene expression across tissues.

We used the classification of 19,709 human protein-coding genes according to their tissue-specific expression presented in the Human Protein Atlas [42]. We found that the compendium was enriched in genes belonging to the *tissue-elevated* category, which comprised all genes assigned to the *tissue enriched*, *group enriched,* and *tissue enhanced* categories (Fig. 4). To identify tissues or organs important for FB/BW regulation, we utilized the TSEA tool [43]. It evaluates the significance of overlaps between genes from the compendium and cell-specific lists of transcripts enriched in a particular human organ or tissue (see *Module network analysis* section).

We found a significant (*p-*value < 0.05) overlap between genes from compendium and the TSEA lists of tissue-enriched transcripts from seven tissues and organs (adipose tissue, adrenal gland, breast, pituitary gland, pancreas, liver, and brain) (Fig. 5). The same analysis was performed for two gene groups (Rank_1: *genes with biological interpretation* and Rank_2: *genes without biological interpretation*) and five gene sets (*Publications, OMIM_allelic_variants, OMIM_all_text, GWAS meta-analysis, Syndromes*). It revealed six more tissues/organs. In the great majority of cases (in the analyses of three gene sets and of the entire compendium), the highest numbers of genes were found in the overlaps with the cell-specific list of genes enriched (pSI < 0.05) in the brain (Fig. 6). This observation confirms the idea that the nervous system plays a critical role in body weight regulation. First, a large portion of genes responsible for body weight regulation are involved in central mechanisms controlling appetite and food intake [22, 29, 32]. Second, a clear enrichment of expression in the brain was found for genes controlling BMI according to GWAS studies [43, 69, 70].

### Networks formed by associations between genes/proteins and module network analysis

At the next step, we reconstructed networks presenting pairwise interactions between genes/proteins of three types: (1) *Experimental*; (2) *Knowledge*; (3) *Homology* (see *Networks formed by associations between genes/proteins* section). Each network involved a unique set of genes (Fig. 7a) and had a unique set of top genes (Fig. 7b, Table 3). That is why we decided to consider networks of all three types in our further analysis.

We recognized clusters (highly interconnected regions) in the networks. Clusters may be of different sorts in different types of networks: (1) Clusters in a protein-protein interaction network (*Experimental*) are often protein complexes and parts of pathways, whereas (2) clusters in a protein similarity network (*Homology*) represent protein families. It was found that *Homology* network included several unconnected subnetworks (Fig. 8) One-third of all proteins involved in this network (50 genes/proteins) were involved into two subnetworks comprising G protein–coupled receptors (*Subnetwork 1* and *Subnetwork 4*) (Additional file 1: Table S8). Thus, module network analysis confirmed our finding obtained from functional annotation (Fig. 2b) that a substantial part of the compendium (nearly one-sixth, or 17%) comprised genes encoding receptors, and G-protein-coupled receptors among them.

One of unconnected subnetworks (*Subnetwork 2*) in the network *Homology* consisted of 17 signal transduction molecules (protein kinases, etc.). In addition: (1) Functional analysis (Fig. 2) showed that 9% of genes from the compendium encoded proteins involved in signal transduction. (2) Sixteen overrepresented signal transduction pathways from KEGG, REACTOME and BIOCARTA were revealed by DAVID for the list of genes Rank_1: *genes with biological interpretation* (Fig. 3). (3) Analysis of PPI network with the MCODE tool revealed a highly scored cluster (and then, extended cluster 3) involving proteins associated with insulin signaling (Fig. 9), a key energy balance signaling pathway [71, 72]. Thus, module network analysis together with functional analysis showed that (1) genes encoding signal transduction molecules constituted a substantial portion of genes in the compendium and (2) signaling molecules formed a dense network of physical and functional associations. According to the pathway analysis performed with DAVID, genes/proteins from overrepresented signaling pathways are involved in BW regulation, supporting the cellular response to well-known regulators of energy homeostasis, such as insulin [72], leptin [73], neurotrophins (BDNF, NGF) [74], adiponectin [75], PPARs [76], POMC [77], NPY [78], ghrelin [79], etc.

A little more than one-fifth of all proteins (22.7%) in the *Homology* network were involved in the subnetworks comprising transcription factors and coregulatory proteins (Additional file 1: Table S8: *Subnetwork 3, Subnetwork 5, Subnetwork 7, Subnetwork 13, Subnetwork 14, Subnetwork 18, Subnetwork 29,* and *Subnetwork 30*). The extended Cluster 2 obtained in the MCODE analysis of the PPI network was also enriched in transcription factors and coregulatory proteins. From the functional analysis (Fig. 2b), we found that transcription factors and coregulatory proteins constituted, respectively, 12% and 2% of all genes in the compendium. Taken together, these observations are indicative of an important role of transcription factors and transcriptional coregulators in BW control. This finding is in a good agreement with our knowledge on the cooperative interactions between transcription factors and other coregulatory proteins that form complicated mechanisms of transcription complex assembly [38, 80–82].

Module network analysis performed with the MCODE tool showed that the largest and the highest-scored cluster in the PPI network included proteins associated with cilium morphogenesis. Primary cilia are microtubule-based cellular organelles protruding 1–50 μm from the apical surface of cell membrane; they perceive sensory cues and process extracellular signaling, important for cell functions [83]. Primary cilia have been recognized as the signaling center for processing a large number of homeostasis and developmental signaling pathways (PDGFRalpha, mTOR, Notch, Hedgehog, Wnt, etc.) [84]. Several recent lines of indirect evidence suggest a possible crosstalk between energy balance signaling and ciliary signaling [56]. Receptors for energy homeostatic neuropeptides, neurotransmitters, and growth factors, for example, NPYR (neuropeptide Y receptor), MCHR (melanin-concentrating hormone receptor), and IGFR (insulin growth factor receptor), are located in the cilium, and they employ the ciliogenesis machinery to transduce signals properly [57]. During adipocyte differentiation of human adipose stem cells, the primary cilium goes through dynamic size modifications. This suggests that the cilium has various functions during adipogenesis [85]. A recent study by Seo S. et al. [86] has shown that proteins distorted in the human ciliary disorder Bardet-Biedl syndrome (BBS proteins) are required for leptin receptor (LepR) signaling in the hypothalamus: (1) Bbs2(−/−), Bbs4(−/−) and Bbs6(−/−) mice are resistant to the action of leptin to reduce food intake and body weight; (2) BBS1 protein physically interacts with the leptin receptor (LepR). In our study, LepR was also found to be involved into the network formed by PPIs between proteins responsible for cilium morphogenesis (Fig. 9, extended cluster 1). These observations prove the key role of primary cilia in energy balance and indicate that they may be involved in energy balance as a signaling center for processing numerous homeostasis and developmental signaling pathways, including the LepR signaling pathway.

We found that genes involved in clusters identified with the MCODE tool and their first neighbors (three extended clusters, Fig. 9) were enriched in genes that were classified to the *Expressed in all* and *Mixed* categories. This observation is in a good agreement with the fact that 17 of 20 top genes that have maximal numbers of neighbors in the *Experimental* network (Additional file 1: Table S7) also belong to the *Expressed in all* and *Mixed* categories. It also indicates that physical interactions between proteins from all three extended clusters revealed in our study (Fig. 9) may be functionally significant and important in a broad range of human tissues and organs.

### The ranking of genes from the GWAS meta-analysis set according to the number of neighbors

Meta-analysis of genome-wide association studies (GWAS) resulted in the identification of hundreds of genetic variants associated with elevated body weight. The compendium presented in this report incorporates 164 lead SNPs and 184 genes mentioned in the GWAS meta-analysis papers (Fig. 1, Additional file 1: Tables S4 and S5). However, in most cases it is not easy to find out how these genetic variants influence body weight. In the current study, 119 genes from the GWAS meta-analysis set (or ~65% of all GWAS genes) were classified to the group Rank_2: *genes without biological interpretation*. That is why we concentrated on the exploration of putative mechanisms involving GWAS genes into BW regulation.

With this task in mind, we examined three networks (*Experimental, Knowledge, Homology*) and ranked GWAS genes according to the number of neighbors in these three networks (Fig. 10, and Additional file 1: Table S10). In our opinion, associations revealed for some GWAS genes from the group Rank_2: *genes without biological interpretation* may serve as an additional proof of their relevance to the system of BW regulation*.*

### Identification of GWAS genes in the brain-specific sublist

Expression analysis confirmed the idea that the central nervous system played a critical role in BW regulation: Employment of the TSEA tool showed that the highest number of genes from the compendium overlapped the tissue-enriched list of genes compiled for the brain (Fig. 6). Therefore, at the next step we created a *brain-specific* sublist comprising 249 genes (see *Sublist of proteins expressed in brain* section*,* and Additional file 1: Table S6). We used this sublist for two tasks: (1) analysis of the intersection between the *brain-specific* sublist and genes from the *GWAS meta-analysis* set and (2) construction of the network *Experimental_brain-specific* and its further analysis.

We revealed 71 genes from the *GWAS meta-analysis* set that were found among 249 genes from the sublist *brain-specific* (Fig. 11). That meant that these 71 GWAS genes were either differently expressed in hypothalamic POMC- or AGRP-expressing neurons of mice fed ad libitum and deprived of food [49] (21 genes), or were determined by TSEA as specific to brain at the pSI threshold = 0.05 (see *Gene expression analysis* section) (37 genes), or were found within both categories (13 genes). As shown in Fig. 11, more than a half of GWAS genes found in the intersection between GWAS genes and the *brain-specific* sublist belonged to the group Rank_2: *genes without interpretation.* Moreover, nine genes (*CBLN1, PCDH9, DOC2A, STXBP6, LRP1B, LRFN2, RALYL, LINGO2,* and *HS6ST3*) that belonged to Rank_2: *genes without interpretation* were found in the intersection of groups *Brain-specific gene according to TSEA* and *DEG according to* [49]. Thus, we conclude that these nine GWAS genes are good candidates for further functional analysis. We found that one of these nine genes (*LRP1B*) belonged to the network *Experimental_Brain-specific*, (Fig. 12), and its presumed functional relevance to BW regulation will be discussed below.

### The subnetwork involving proteins expressed in brain: search for GWAS genes

At the final step, we examined the brain-specific network of genes/proteins. This network encompassed physical interactions between proteins from the *brain-specific* sublist (Additional file 1: Table S6). The *Experimental_brain-specific* network involved 117 genes from the compendium. Among them, 22 genes were from the *GWAS meta-analysis* set (Additional file 1: Table S11). Six GWAS genes (*ETV5, LRP1B, NDUFS3, SNRPC, ERBB4,* and *PRKD1)* of these 22 belonged to the group Rank_2: *genes without biological interpretation*. For three PPIs (between ETV5 and AR, LRP1B and SERPINE1, NDUFS3 and ADRB2), the quality of evidence for direct physical interactions was very high (Table 4). Therefore, it is highly likely that *ETV5, LRP1B, NDUFS3* are involved in the brain-specific network. Associations involving three other genes (*SNRPC, ERBB4, PRKD1*) may also take place, as follows from the evidence for PPIs found in other species. In addition, SNRPC had two PPIs (with TOMM40 and SNRPN), confirming the idea that this gene/protein was involved in the *Experimental brain-specific* network.

**Fig. 13:**
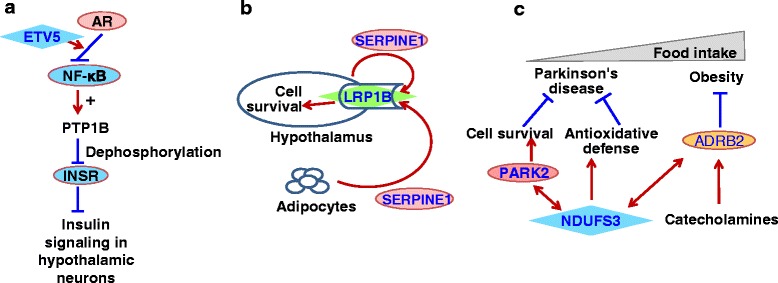
Putative regulatory pathways involving physical interactions revealed within the network *Experimental_brain-specific*: Panel **a**. Interactions between ETV5 and AR. Panel **b**. Interactions between LRP1B and SERPINE1. Panel **c**. The regulatory scheme involving NDUFS3, PARK2, and ADRB2. Ellipses indicate proteins/genes from the group Rank_1: *genes with biological interpretation.* Lozenges indicate proteins/genes from the group Rank_2: *genes without biological interpretation*. Genes/proteins from the *GWAS meta-analysis* set are shown in blue

Thus, we suppose that these six GWAS genes (*NDUFS3 SNRPC ERBB4, ETV5, PRKD1,* and *LRP1B*) participate in BW regulation via PPIs with proteins whose biological roles in BW regulation are already known. On the base of the most confident PPIs, we propose three putative regulatory schemes:

The first regulatory scheme (Fig. 13a)

The androgen receptor (AR) belongs to the superclass of nuclear receptors. It serves as a negative regulator of adipose tissue development in adult males: an increase in the wet weights of white adipose tissues was found in 30-week-old male androgen receptor knockout mice [87]. In the brain, particularly, in the hypothalamus, ARs appear to participate in the regulation of insulin sensitivity and glucose homeostasis [88]. Experiments with AR-deficient mice demonstrated that the loss of neuronal AR led to increased activation of hypothalamic nuclear factor κB (NF-κB), which induces the expression of protein-tyrosine phosphatase 1B (PTP1B) [89]. In turn, PTP1B interferes with hypothalamic insulin signaling via insulin receptor (INSR) dephosphorylation [90].

*ETV5* was found to be one of the top 30 genes differentially expressed in POMC-expressing neurons of mice fed ad libitum and deprived of food [49]. According to [91], ARs may negatively regulate the transcriptional activity of target genes through protein-protein interactions with the other transcription factor, ETV5, rather than through direct specific interaction with promoter DNA. Thus, ETV5 may participate in the regulatory mechanism involving NF-κB downregulation and subsequent decline in PTP1B activity. In turn, decreased PTP1B activity may affect the rate of insulin receptor dephosphorylation and thereby impair insulin signaling in hypothalamic neurons (Fig. 13a).

The second regulatory scheme (Fig. 13b)

The *SERPINE1* gene encodes serpin family E member 1, also known as endothelial plasminogen activator inhibitor-1 (PAI1). PAI1/SERPINE1 is an adipokine, a member of the serine protease inhibitor family. Adipocytes are the main contributor to the elevated levels of PAI-1/SERPINE1 seen in obesity. However, PAI1/SERPINE1 is also produced within the brain by neurons, astrocytes and microglia, and it may regulate migration of microglia, survival of neurites, and apoptosis [92]. *PAI1/SERPINE1* was found to be differentially expressed in mouse hypothalamic neurons according to [41] (Sublist *Brain-specific,* *Sublist of proteins expressed in brain* section, and Additional file 1: Table S6). With regard to the function of PAI1/SERPINE1 in a non-obese hypothalamus, it is pertinent to mention that transgenic mice overexpressing urokinase-type plasminogen activator (uPA) in the paraventricular nucleus of the hypothalamus and some other brain regions also exhibit reduced body weight and energy intake [93]. PAI1/SERPINE1 is considered the primary inhibitor of uPA. Thus, overexpression of PAI1/SERPINE1 within the hypothalamus can attenuate expression of uPA and, as a result of thereof, increase energy intake and body weight, which are the main parameters in obesity [92]. PAI1/SERPINE1 prevents the disintegration of formed neuronal networks by promoting or maintaining neuroprotective signaling through the MAPK(ERK) pathway [94]. PAI1/SERPINE1 activates microglial cells through the LRP1/JAK/STAT1 axis and promotes migration of microglial cells in culture [95].

LDL receptor related protein 1B (*LRP1B*) is a member of the low-density lipoprotein receptor gene family. It has been shown that PAI1/SERPINE1 is a ligand for LRP1B [96]. On the cell surface, LRP1B binds and internalizes PAI1/SERPINE1, mediating the function of the urokinase plasminogen activator/receptor [96]. *LRP1B* is expressed in the whole brain specifically according to TSEA and is a DEG in murine POMC- or AGRP-expressing hypothalamic neurons [49] as well (Fig. 11, and Additional file 1: Table S6). Therefore, we hypothesize that LRP1B mediates the effect of PAI1/SERPINE1 on hypothalamic neurons, promoting cell survival and leading to increased energy intake (Fig. 13b). This regulatory loop may involve: (1) PAI1/SERPINE1 produced by adipocytes, and (2) PAI1/SERPINE1 produced within the brain by neurons, astrocytes and microglia as well, where it mediates paracrine and autocrine signaling between hypothalamic cells. In such a way, the interaction between LRP1B and PAI1/SERPINE1 may contribute to the crosstalk between central and peripheral signals produced within the brain and by adipocytes respectively.

The third regulatory scheme (Fig. 13c).

*ADRB2* encodes beta-2 adrenoceptor, which is a major lipolytic receptor in human fat cells [97]. ADRB2 is involved in the regulation of the catecholamine function, and it may be of particular importance for human obesity because of the central role of catecholamines in energy expenditure. Loss of noradrenergic terminals in ventral bundle termination areas, e.g., the hypothalamus, may lead to hyperphagia in mice [98]. OMIM contains convincing proofs of associations between *ADRB2* allelic variants and obesity. In mice, *ADRB2* is differentially expressed in hypothalamic neurons, which confirms its involvement in the central regulation of feeding behavior. Also in mice, *ADRB2* demonstrates more than tenfold difference in expression level between AGRP- and POMC-expressing neurons in the fed group [49].

NADH:ubiquinone oxidoreductase core subunit S3 (*NDUFS3*) encodes one of the iron-sulfur protein components of mitochondrial NADH:ubiquinone oxidoreductase (complex I). At present, we have no idea concerning potential molecular mechanisms effecting the central regulation of energy homeostasis (or BW) and involving physical interactions between ADRB2 and NDUFS3. A small piece of evidence indirectly confirming the idea that NDUFS3 affects FB was found in the KEGG pathways database. According to KEGG, NDUFS3 is involved in molecular events associated with Parkinson disease (KEGG Pathway ID = hsa05012 - Parkinson’s disease - Homo sapiens). NDUFS3 is a component of the first enzyme complex in the electron transport chain of mitochondria. Cleavage of NDUFS3 disrupts mitochondrial metabolism and generates reactive oxygen species (ROS), which trigger the programmed cell death pathway [99]. On the other hand, weight loss has also been reported in advanced stages of Parkinson’s disease. These weight changes are multifactorial. They involve changes in energy expenditure, perturbation of homeostatic control, and feeding behavior. [100]. Thus, we assume that interactions between NDUFS3 and ADRB2 may participate in the crosstalk between catecholamine signaling and antioxidative defense mechanisms that provide neuronal longevity and activity (Fig. 13c).

Parkin RBR E3 ubiquitin protein ligase, also known as PARK2, was the second protein found to be the first neighbor for NDUFS3 in the *Experimental_brain-specific* network (Fig. 12)*.* The enzyme is a component of the multiprotein E3 ubiquitin ligase complex that mediates the targeting of substrate proteins for proteasomal degradation. PARK2 regulates cell proliferation or cell survival [101]. In mice, E3 ligase Park2 is upregulated in AGRP-expressing neurons during food deprivation [49]. The quality of evidence confirming interactions between NDUFS3 and PARK2 was not high (Table 4). Nevertheless, PARK2 involvement in autosomal recessive juvenile Parkinson disease [102] confirms the regulatory scheme presented in Fig. 13c.

## Conclusions

Obesity is a complex disorder, involving multiple genes and multiple biological processes and physiological systems in the human body. The main objectives of the present study were: (1) to collect as full as possible list of genes involved in FB and BW regulation and to formalize it as a compendium; (2) to obtain functional characteristics of genes by using different theoretical approaches and to create a catalog of biological processes, biochemical/signaling pathways, and organs/tissues important for the regulation of BW and FB; and (3) to rank GWAS genes.

At present, the compendium contains 578 human genes for which there are indications of their direct or indirect relevance to the regulation of feeding behavior or body weight.

Here we present functional characteristics of genes regulating FB and BW that were revealed based on the compendium by using several complementary theoretical approaches. We admit that none of them can objectively define the most important feature of genes. Nevertheless, we found that the integration of approaches was useful because the combined result demonstrated the complexity and hierarchy of FB and BW regulation.

**Fig. 14:**
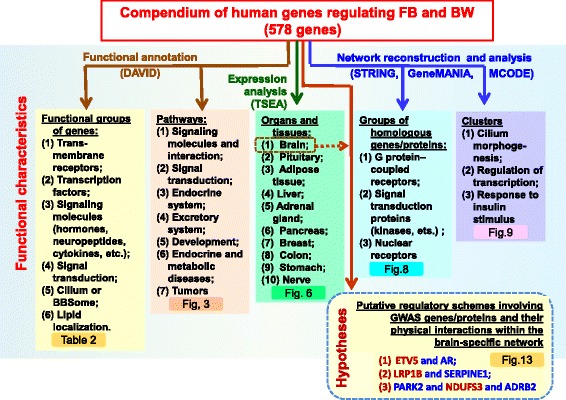
A catalog of functional characteristics of genes regulating BW and FB revealed in the current study. Here we present only the most important molecular functions, biochemical/signaling pathways, organs/tissues, and biological processes: (1) non-overlapping functional groups of genes that were overrepresented in the compendium; (2) the classification of enriched pathways performed using the hierarchical scheme provided by the KEGG pathway database (the full list of pathways is presented in Fig. 3 and Additional file 2: Figure S1); (3) TSEA organs and tissues with the cell-specific lists of tissue-enriched transcripts/genes that were overrepresented with genes from the group Rank_1: *genes with biological interpretation*; (4) homologous groups of proteins including more than three proteins; (5) extended clusters with scores of initial clusters exceeding 3.3

On the results of our complex analysis, we revealed and catalogued molecular functions of encoded proteins, biological processes, and biochemical or signaling pathways enriched in genes from the compendium. We outlined a group of tissues and organs important for FB and BW control (Fig. 14). We analyzed networks formed by associations between genes/proteins from the compendium and revealed notable clusters formed by G protein-coupled receptors and nuclear receptors, as well as extended clusters of genes involved in the following basic intracellular processes: cilium morphogenesis, transcription regulation, and insulin signaling. We analyzed expression data from the Human Protein Atlas [42] and concluded that physical interactions between proteins involved in extended clusters associated with basic intracellular processes may be functionally significant in a broad range of human tissues and organs. Thus, on the base of module network analysis we organized data on functional groups of genes from compendium that orchestrate the biological activities of different cell types from different tissues and organs according to the demands of the whole human body (Additional file 1: Tables S5, S8, S9).

The expression analysis demonstrated that the highest number of genes from the compendium belonged to the brain list of tissue-enriched genes. Thus, our result confirms the widespread opinion that the central nervous system plays a critical role in body weight regulation. In view of this fact, we reconstructed the brain-specific network formed by physical interactions among genes/proteins from the compendium. Resting on the analysis of the brain-specific network, we proposed potential mechanisms involving three GWAS genes (*ETV5*, *LRP1B*, and *NDUFS3*) in body weight regulation.

**Fig. 15:**
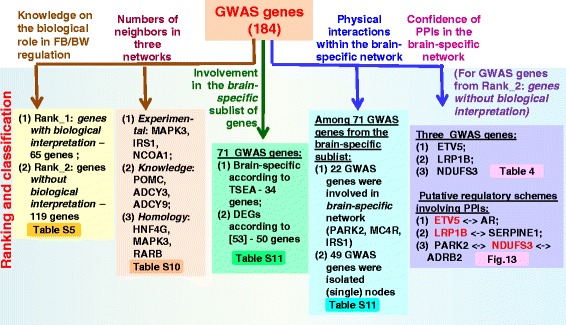
Ranking and classification of GWAS genes from the compendium according to various criteria. For ranking based on the number of neighbors in three networks, we present three top genes for each network. For GWAS genes involved in the *brain-specific* network, three top genes (selected according to the number of neighbors) are presented

The assortment of genes in the compendium provided us with the possibility to prioritize genes from the *GWAS meta-analysis* set. Prioritization of the GWAS genes is a distinct and important problem due to the lack of information on the biological roles of many GWAS genes in BW control. We performed five ranking procedures (Fig. 15) taking into account: (1) knowledge on the biological role in FB/BW regulation; (2) the numbers of neighbors in three networks; (3) the involvement in the *brain-specific* sublist of genes; (4) the numbers of physical interactions within the *brain-specific* network; and (5) the confidence of PPIs in the *brain-specific* network (this procedure was performed for GWAS genes from the group Rank_2: *genes without interpretation*).

Here we present the most comprehensive catalog of functional characteristics of genes controlling body weight than those published before in the most recent studies [5, 29]. Use of different sources of data (*Publications, OMIM*, and *GWAS*) allowed us to select and analyze more than five hundred genes. As a result, we (1) formed a more complete list of tissues and organs important in body weight control (aside from the brain or parts of the central nervous system) and (2) distinguished a unique combination of functional characteristics of proteins notable for body weight regulation. The framework formed by these proteins (G protein-coupled receptors, cilium and BBSome components, transcription factors, and proteins involved in insulin signaling) regulates the biological activities of particular cells in accordance with the energy state at the whole-body level.

Our analyses can be regarded as a new step towards a comprehensive list of genes, tissues/organs, biological processes, and pathways involved in FB and BW regulation. It may provide grounds for the development of more holistic disease models and new therapeutics, which is one of the major concerns of obesity research.

## Additional files

Additional file 1: Table S1. Human genes (105) controlling feeding behavior that were collected from scientific publications (research papers and review articles); **Table S2.** Human genes (336) controlling feeding behavior or body weight that were collected from OMIM; **Table S3.** Human genes (37) associated with syndromes that include *obesity* as one of the phenotypic characteristics (Bardet-Biedl, Prader-Willi, Alstrom, etc); **Table S4.** Data from the GWAS meta-analysis papers. Lead SNPs with genome-wide significant (*p-*value < 5 × 10^−8^) associations with BMI (164); and genes listed in GWAS meta-analysis articles and located in the regions around the 164 lead SNPs (184). **Table S5.** All genes from the compendium (578 genes); **Table S6.** The brain-specific sublist of genes (column C) that was obtained by merging two lists (presented in columns A and B); **Table S7.** Top twenty genes that have the maximal numbers of the first neighbors in each network (*Experimental, Knowledge, Homology*) and their main characteristics; **Table S8.** Unconnected subnetworks found within the network *Homology*; **Table S9.** Top-scoring GO terms from the chart GOTERM_BP_5 obtained with the DAVID tool for three lists of genes. The lists of genes analyzed here were obtained by extending three top-scoring clusters revealed in the protein interaction network *Experimental* with the MCODE tool (see *Module network analysis* section). Only GO terms with FDR below E-07 are presented; **Table S10.** Genes from the GWAS meta-analysis set that are involved in three networks (*Experimental, Knowledge, Homology*) and have at least one neighbor; **Table S11.** Genes involved in the brain-specific network of PPIs (*Section A*) and single (isolated) genes (*Section B*) that belong to the sublist *brain-specific*. (XLSX 188 kb)
Additional file 2: Figure S1. Association of genes from the compendium with major KEGG, REACTOME and BIOCARTA pathways. Pathways with fold enrichment > 1.5 and BH adjusted *p-*value < 0.05 are presented. This figure presents the results for the sets of genes *Publications, OMIM_allelic_variants, OMIM_all_text, Syndromes* and *GWAS meta-analysis*. (DOCX 600 kb)

## Abbreviations

BMI: Body mass index
BW: Body weight
DEG: Differentially expressed gene
eQTL: Cis-expression quantitative trait locus
FB: Feeding behavior
GWAS: Genome-wide association studies
LD: Linkage disequilibrium
PPI: Protein-protein interaction
SNP: Single nucleotide polymorphism
TF: Transcription factor
