# Supplementary material for: A compendium of human genes regulating feeding behavior and body weight, its functional characterization and identification of GWAS genes involved in brain-specific PPI network
Source: BMC Genet. 2016 Dec 22;17(Suppl 3):158. doi: 10.1186/s12863-016-0466-2 (PMC5249002; doi:10.1186/s12863-016-0466-2)
Supplement: Additional file 2: Figure S1. — Association of genes from the compendium with major KEGG, REACTOME and BIOCARTA pathways. Pathways with fold enrichment > 1.5 and BH adjusted p-value < 0.05 are presented. This figure presents the results for the sets of genes Publications, OMIM_allelic_variants, OMIM_all_text, Syndromes and GWAS meta-analysis. (DOCX 600 kb) [file 12863_2016_466_MOESM2_ESM.docx]

**Additional file 2**

**For the manuscript**

**A compendium of human genes regulating feeding behavior and body weight, its functional characterization and identification of GWAS genes involved in brain-specific PPI network. (**Ignatieva E.V., Afonnikov D.A., Saik O.V., Rogaev E.I., and Kolchanov N.A.)

| 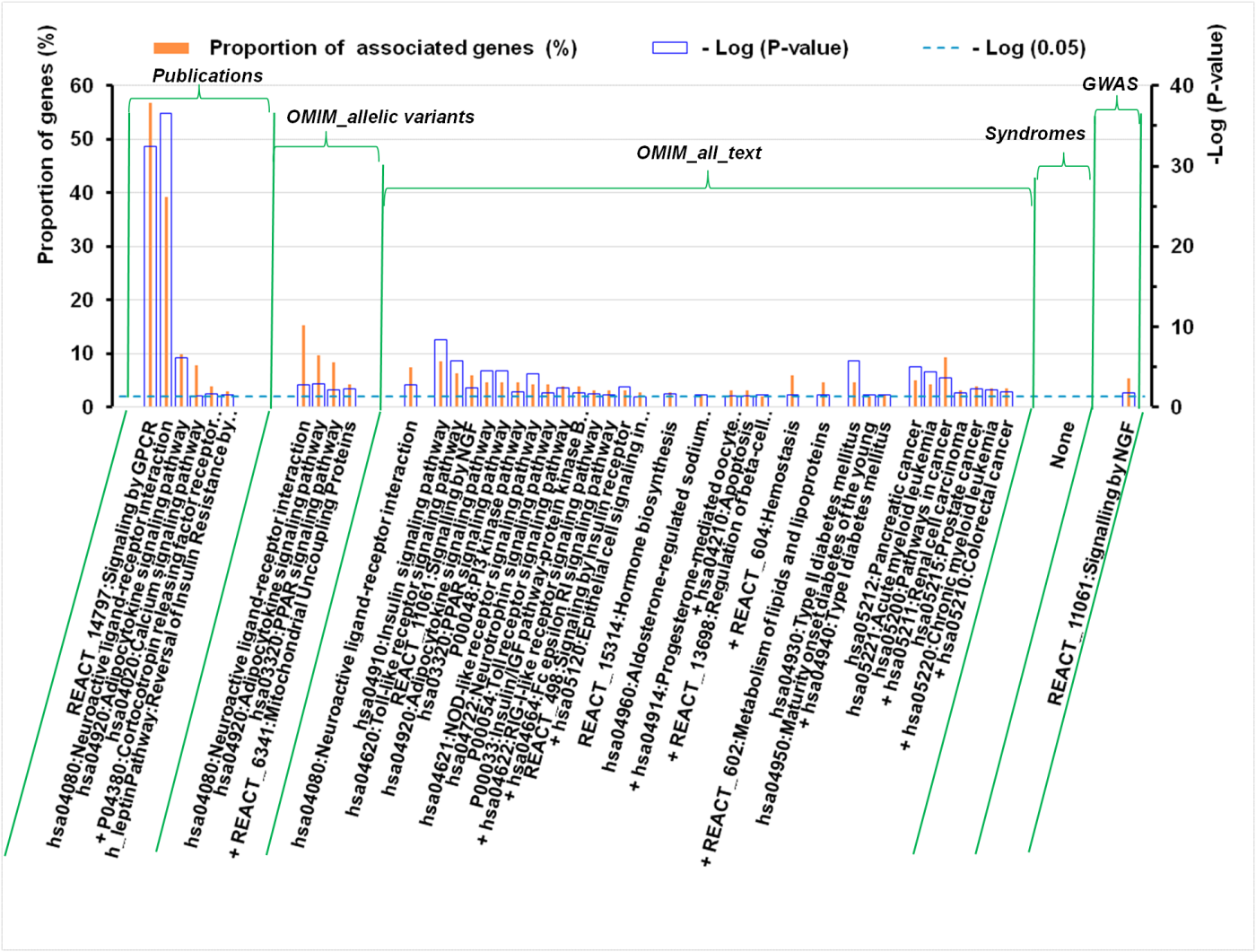 |
| --- |
| Figure S1. Association of genes from the compendium with major KEGG, REACTOME and BIOCARTA pathways. Pathways with fold enrichment > 1.5 and BH adjusted p-value < 0.05 are presented. This figure presents the results for the sets of genes *Publications, OMIM_allelic_variants, OMIM_all_text, Syndromes* and *GWAS_meta-analysis*. Pathways not found to be enriched for the subset Rank_1: *genes with biological interpretation* (presented in Fig. 3) are marked by plus signs. |
